# Small Molecule Inhibitors of AI-2 Signaling in Bacteria: State-of-the-Art and Future Perspectives for Anti-Quorum Sensing Agents

**DOI:** 10.3390/ijms140917694

**Published:** 2013-08-29

**Authors:** Min Guo, Sonja Gamby, Yue Zheng, Herman O. Sintim

**Affiliations:** Department of Chemistry and Biochemistry, University of Maryland, Building 091, College Park, MD 20742, USA; E-Mails: mguo@umd.edu (M.G.); sonja.gamby@gmail.com (S.G.); yuezheng@umd.edu (Y.Z.)

**Keywords:** quorum sensing, virulence, biofilm formation, bacteria, autoinducer, antagonists, AI-2

## Abstract

Bacteria respond to different small molecules that are produced by other neighboring bacteria. These molecules, called autoinducers, are classified as intraspecies (*i.e*., molecules produced and perceived by the same bacterial species) or interspecies (molecules that are produced and sensed between different bacterial species). AI-2 has been proposed as an interspecies autoinducer and has been shown to regulate different bacterial physiology as well as affect virulence factor production and biofilm formation in some bacteria, including bacteria of clinical relevance. Several groups have embarked on the development of small molecules that could be used to perturb AI-2 signaling in bacteria, with the ultimate goal that these molecules could be used to inhibit bacterial virulence and biofilm formation. Additionally, these molecules have the potential to be used in synthetic biology applications whereby these small molecules are used as inputs to switch on and off AI-2 receptors. In this review, we highlight the state-of-the-art in the development of small molecules that perturb AI-2 signaling in bacteria and offer our perspective on the future development and applications of these classes of molecules.

## 1. Introduction

### 1.1. A Paradigm Shift from Bactericidal and Bacteriostatic Agents to Anti-Virulence Agents

**Figure 1:**
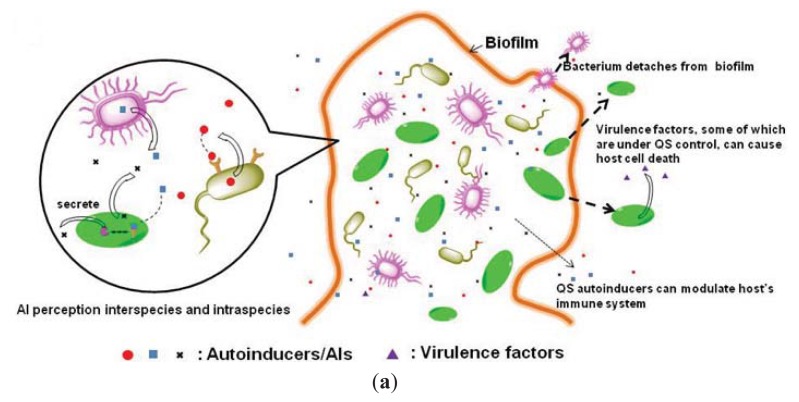

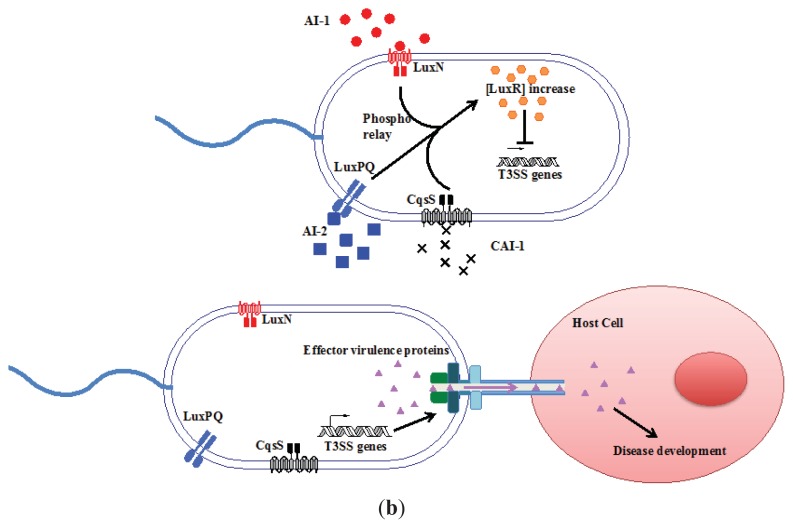
(**a**) Increased concentration of autoinducers in bacterial biofilms promotes the synthesis of biofilm matrices, such as adhesion proteins and polysaccharides, which are required for the maintenance of the biofilm structure; (**b**) Autoinducers repress the production of virulence factors as well as the synthesis of the components of the bacterial secretory system, such as T3SS, in some bacteria (for example, AI-1, AI-2 and CAI-1 represses T3SS gene expression in *V. harveyi* [12]).

Bacteria have developed sophisticated mechanisms to render almost any antibiotic harmless. There is a paucity of newly approved US Food and Drug Administration (FDA) antibiotics and the newly approved antibiotics share similar chemical motifs to existing drugs, meaning that these so-called newer drugs would ultimately succumb to bacterial resistance [1–3]. Bacteriostatic or bactericidal drugs put evolutionary pressure on pathogens to develop resistance and so a new strategy to treat bacterial infections, which does not involve the killing of bacteria but rather curb bacterial virulence, is needed [4]. It is evident that bacterial virulence production and biofilm formation are sometimes controlled by quorum sensing (QS, see Figure 1) [5–7], a system bacteria use to communicate and respond as a collective but not critical to individual vitality. Bacterial QS was discovered almost half a century ago. In 1965, a hormone-like cell product was discovered in *S. pneumoniae* [8] and was later identified as autoinducing peptide [9]. The term quorum sensing was first defined by Nealson and co-workers in 1970 to describe the production of light by *V. fischeri* at high cell densities (*i.e*., a population-dependent process) [10,11]. Nealson and co-workers then postulated that the bioluminescence from *V. fischeri* was regulated by molecules, called “autoinducers”. Subsequently, several autoinducers (both intraspecies and interspecies) have been identified.

Due to the pivotal role played by quorum sensing in bacterial pathogenesis (virulence expression) and resistance (biofilm formation), quorum sensing receptors have emerged as potential targets for anti-infective therapy.

**Figure 2:**
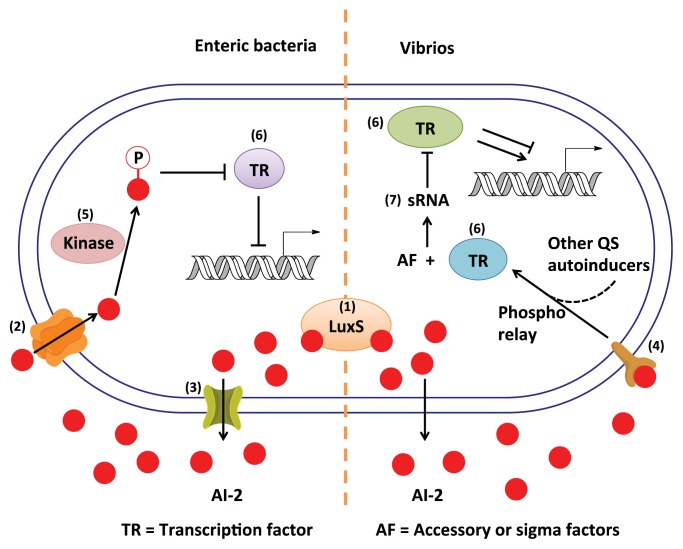
Possible AI-2-based druggable targets. (**1**) LuxS; (**2**) AI-2 transporter (such as LsrB); (**3**) efflux pump for AI-2; (**4**) extracellular receptor for AI-2 (such as LuxP); (**5**) intracellular receptor for AI-2; (**6**) AI-2-regulated transcription factor or repressor (such as LsrR); (**7**) small regulatory RNA (sRNA) mediated quorum sensing (QS) circuit.

Because autoinducers (AIs) are the signaling molecules in QS, one can reasonably assume that antagonists of AIs would reduce toxin production and biofilm formation in some bacteria. It is however worth mentioning that bacterial toxin production and biofilm formation could also be regulated by other pathways, other than QS, so anti-QS agents should not be considered as panacea for reducing all toxin production and biofilm formation. There are three major classes of autoinducers (Figure. 2): AI-1 (AHLs) [13], oligopeptides/AIP (autoinducing peptide) [9,14] and AI-2 [15]. There are also other bacterial signaling molecules that do not fall under the above three classes, such as PQS (*P. pseudomonas* quinolone signal, **2**) [16], γ-butyrolactone [17,18], CAI-1(**5**) [19,20], DSF (diffusible signal factor, **6**) [21], 2-AA (2-amino acetophenone, **7**) [22], DKP (diketopiperazine, **8**) [23], IQS (**10**) [24] and CSP (competence stimulating peptide, **9**) [25]. Except AI-2, which is the term for interconverting equilibrium mixture of compounds derived from DPD, other AIs are species-specific. For example, AI-1 mediates species-specific Gram-negative bacteria QS [26], oligopeptides are found in Gram-positive bacteria [27], PQS is one of the QS signaling molecules in *P. aeruginosa* [28], and CAI-1 is produced by vibrios [29]. Interestingly, AI-2, which is found in many (~70) species of both Gram-negative and Gram-positive bacteria, is an interspecies autoinducer and goes by the moniker “universal autoinducer” [30]. Plausibly, AI-2 inhibitors could have broad spectrum anti-quorum sensing properties and be used in synergy with other antibiotics [31]. For phenotypes that are regulated by the AI-2/LuxS system, the reader is referred to an excellent review by Xavier *et al.* [32] and Table 1.

### 1.2. Inhibition of Quorum-Sensing as an Anti-Virulence Strategy

In the last decade, attempts have been made to find or develop inhibitors for different receptors (shown in Figure 2), which are involved in the production and perception/response to AI-2. This review aims to highlight the current understanding of AI-2 signaling in bacteria and provide examples of small molecules, which have been shown to inhibit AI-2 signaling in bacteria. For excellent reviews on the inhibition of signaling by other autoinducers, see those by Blackwell, Spring or Federle [46–48].

## 2. Synthesis of AI-2

### 2.1. Biosynthesis of AI-2

**Figure 3:**
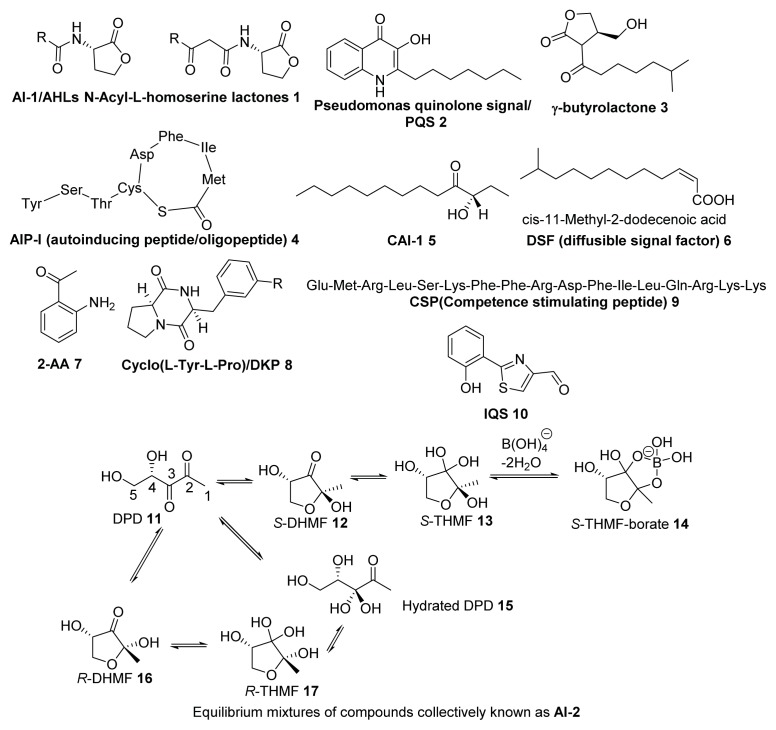
Autoinducer molecules. AI-2 is a term used to described DPD and isomers in equilibrium [49,56].

**Scheme 1:**
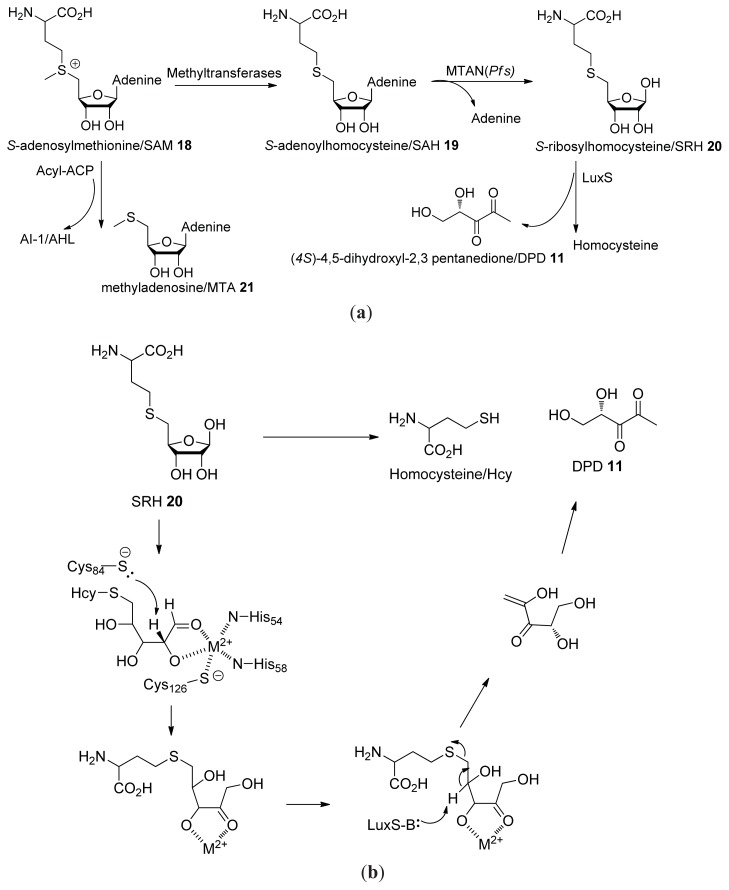
(**a**) Biosynthetic route to DPD; (**b**) Mechanism of LuxS-catalyzed transformation of SRH into DPD (Adapted from [55] with permission. Copyright 2009, Elsevier).

The primary biosynthetic route to AI-2 in bacteria has been established as the LuxS-catalyzed production of 4,5-Dihydroxy-2,3-pentanedione (DPD) from *S*-ribosyl-l-homocysteine/SAH (**19**, Scheme 1) [30,49–51]. Upon the formation of DPD, it spontaneously cyclizes into different isomers that are in equilibrium with each other (see Figure 3). LuxS homologues exist in about 50% of all sequenced bacteria (both Gram-negative and Gram-positive) [52]. It is worth noting that LuxS is a dual function enzyme, which not only produces AI-2 but also participates in activated methyl cycle (AMC). Hence, it is still a matter of debate whether AI-2 is a bona fide AI or just a metabolite in AMC [53].

**Scheme 2:**
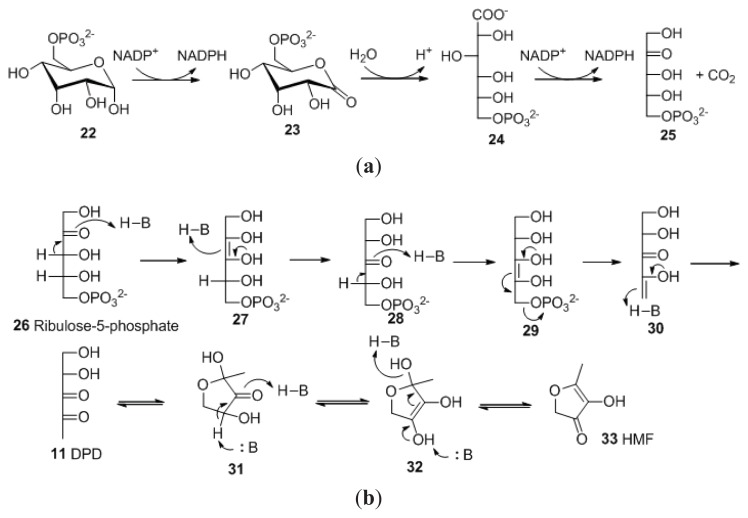
(**a**) Generation of d-ribulose-5-phosohate in the OPP pathway; (**b**) Degradation pathway of Ru5P to form 4,5-dihydroxy-2,3-dipentadione and HMF.

Another pathway to AI-2, which does not involve the activated methyl cycle, has also been proposed. It has been shown that in the presence of acid, both DPD and 4-hydroxy-5-methyl-3(2*H*)-furanone (HMF, **33**) spontaneously form from d-ribulose-5-phosphate (Ru5P, **26**) (Scheme 2b) [54]. MHF has been shown to have moderate effects on bioluminescence in *V. harveyi* [50]. Ru5P is formed during the catabolism of glucose via the oxidative pentose phosphate (OPP) pathway (Scheme 2a).

Using an *E. coli* mutant, which degrades glucose exclusively through the OPP pathway, Tavender and coworkers showed that culture supernatants had modest activity in a *V. harveyi* bioassay [57]. This suggested that DPD had been generated *via* an alternative, non-enzymatic, pathway. It has been suggested that in some species that lack LuxS, such as the Oomycetes *Phytophthora* and *Pythium*, Ru5P could be a good source of AI-2 [43]. Kong and coworkers have shown that supernatants from these bacteria, lacking *luxS*, could stimulate an AI-2-mediated response (bioluminescence) in *V. harveyi* [58]. Nichols and coworkers have also demonstrated Ru5P as a LuxS independent source of DPD in the thermal-resistant bacteria, *T. maritima* [59]. While *T. maritima* produced AI-2, it did not respond to that which was exogenously supplied. AI-2 may serve as metabolic byproduct in some species but there is much evidence supporting its role in the repression and activation of a wide range of genes [60]. Therefore, there are interests in small molecules that can antagonize the biological effects of AI-2 in bacteria.

### 2.2. Chemical Synthesis of AI-2

**Scheme 3:**
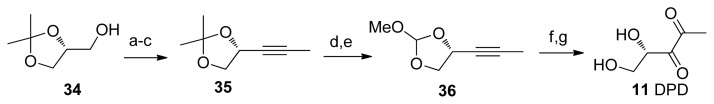
First reported synthesis of DPD and analogs. Reagents and conditions: (**a**) Oxayl chloride, DMSO, CH_2_Cl_2_; Et_3_N; (**b**) Zn, CBr_4_, Ph_3_P, CH_2_Cl_2_; (**c**) *t*-BuLi, CH_3_I, THF; (**d**) 60% acetic acid; (**e**) CH(OMe)_3_ (neat), H_2_SO_4_ (cat); (**f**) KMnO_4_, acetone buffer (aq); (**g**) H_2_O, pH 6.5 (K_2_HPO_4_/KH_2_PO_4_ (0.1 M), NaCl (0.15 M)), 24 h.

Unlike AI-1, for which several groups have reported various analogs that are effective QS inhibitors, the development of AI-2-like analogs that have biological effects had lagged behind until the recent works of Janda [61–64], Sintim [65–68], Doutheau [69], Meijler [70] and Ventura [71]. Over the last decade there have been numerous reported syntheses of AI-2 and AI-2 analogs. Notably, the first chemical synthesis of AI-2 was accomplished by Janda and co-workers in 2004 (Scheme 3) [61]. Janda’s synthesis started from commercially available alcohol **34**, which was then oxidized into an aldehyde using Swern oxidation, followed by Corey-Fuchs homologation to afford acetal protected alkyne **35**. Compound **35** was then deprotected and converted into orthoformate **36 (**73% yield over two steps). The orthoformate is easier to deprotect, using milder acidic conditions, than the acetal. After oxidation of alkyne **36** by KMnO_4_ (10% yield) and deprotection in weak acidic buffer (quantitative yield), s-DPD was obtained *in situ*. This first synthesis of DPD involved seven steps to give *S*-DPD in an overall yield of 3.2%. Variations of Janda’s synthesis were later published by other groups, with the difference being either (1) selection of protection groups [72] or (2) route to the diketone moiety. [73]

**Scheme 4:**
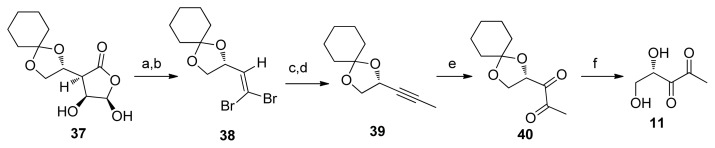
Semmelhack’s synthesis of DPD [72]. Reagents and conditions: (a) KIO_4_, K_2_CO_3_, H_2_O/CH_2_Cl_2_; (b) Ph_3_P, CBr_4_; (c) 1. *n*-*Bu*Li, 2. H_2_O; (d) 1. *n-*BuLi, 2. CH_3_I; (e) RuO_2_ (cat.), NaIO_4_; (f) pH 1.5.

Shortly after Janda’s synthesis, Semmelhack also published a synthesis of AI-2 from the monocyclohexylidene derivative of l-gulonic acid γ-lactone (**37**, Scheme 4). [72] Semmelhack improved Janda’s synthesis of DPD by introducing a cyclohexylidene protecting group (there was no need for a protecting group interchange in this case). Compound **37** in Semmelhack’s synthesis was prepared in 75% yield from readily available l-gulonic acid γ-lactone, and then converted to an aldehyde by KIO_4_ (78% yield). Following a similar Corey-Fuchs homologation, alkyne **39** was obtained in a 43% yield over two steps. In Janda’s synthesis, the low overall yield was mainly due to the poor yield obtained during the KMnO_4_ oxidation (10%). Semmelhack therefore utilized a RuCl_2_-catalyzed NaIO_4_ oxidation of alkyne **39** to afford diketone **40** in a much more improved yield (70%), compared with KMnO_4_. An uneventful acidic deprotection of compound **40** afforded *S*-DPD in an overall yield of 24%.

**Scheme 5:**
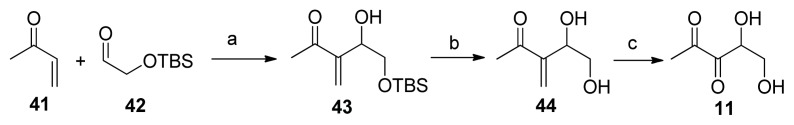
Doutheau’s synthesis of DPD. Regents and conditions: (**a**) THF, DABCO, 0 °C; (**b**) TBAF/THF, RT; (**c**) 1. O_3_, MeOH, −78 °C; 2. DMS, −78 °C to RT.

**Scheme 6:**
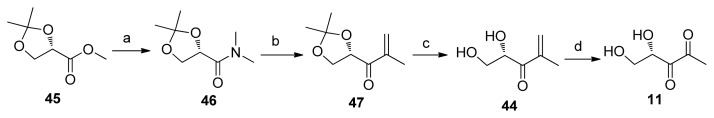
Vanderleyden’s synthesis of DPD. Reagents and conditions: (**a**) NH(CH_3_)_2_, EtOH; (**b**) CH_2_=CCH_3_MgBr, Et_2_O/THF; (**c**) Dowex resin, MeOH; (**d**) O_3_, MeOH, DMS.

Doutheau published a short, three-step synthesis of AI-2 based on the Baylis-Hilman reaction (Scheme 5) [74]. Commercially available silyl protected aldehyde **42** and enone **41** afforded Baylis–Hilman product **43** in 74% yield. **43** was then deprotected by TBAF to give diol **44** in a 78% yield. Compound **44** was then subjected to reductive ozonolysis, using dimethyl sulfide, to give racemic DPD in an overall yield of 58%. Vanderleyen and co-workers reported a convenient synthesis of AI-2 starting from commercially available acetal ester **45**, which was transformed into an olefin **47** in two steps (Scheme 6). Hydrolysis of the dioxolane ring in **47** on an acidic Dowex resin to give α,β-unsaturated carbonyl **44**, followed ozone-mediated cleavage of the double bond to give DPD (Scheme 6) [73].

**Scheme 7:**
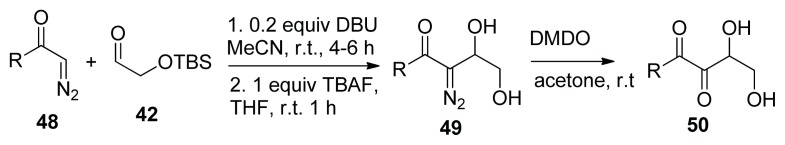
Synthesis of DPD and analogs developed by Sintim and co-workers. DBU = 1,8-diazabicycloundec-7-ene, TBAF = *tert*-butyl ammonium fluoride, THF = tetrahydrofuran, DMDO = dimethyldioxirane.

The Sintim group developed a facile, two-flask synthesis of AI-2, which is amenable to the generation of a variety of C1 AI-2 analogs (Scheme 7) [65]. The key step in Sintim’s synthesis is the Aldol condensation between various diazocarbonyls **48** and a commercially available 2-(*tert*-butyldimethylsiloxy) acetaldehyde **42**. The diazocarbonyls **48**, which are used in Sintim’s synthesis, could be obtained from the requisite acid chloride and diazomethane.

These diazocarbonyls **48** were then condensed with 2-(*tert*-butyldimethylsiloxy) acetaldehyde **42** to afford diazo diol intermediates **49**, after deprotection of the silyl group with *tetra*-butyl ammonium fluoride. Column chromatography purification of the diazo diol followed by oxidation with dimethyl dioxirane resulted in pure racemic DPD and analogs **50** in moderate to high yields (up to 39% overall yield). Potentially, enantioselective diazo Aldol reactions, developed by Trost [75] and others [76] could be adopted to make enantio-enriched DPD and analogs, using Sintim’s methodology. To date, Sintim’s synthesis has produced many C1-modified AI-2 analogs (with linear, branched, cyclic, and, aromatic C1 groups).

**Scheme 8:**
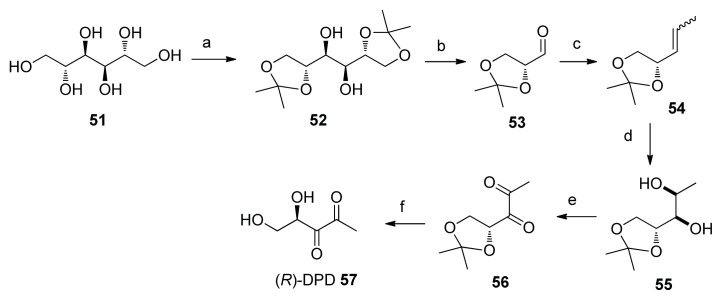
Synthesis of *R-* and *S*-DPD by Gardiner and co-workers: (**a**) 2,2-dimethoxypropane, *p*-TSA, 57%; (**b**) NaIO_4_, NaHCO_3_, 75%; (**c**) (ethyl)triphenyl phosphonium bromide, *n*-BuLi, 70%; (**d**) 4% OsO_4_, NMO·H_2_O, 70%; (**e**) PCC, 30%; (**f**) H_2_O, H_2_SO_4_.

Gardiner and co-workers reported a new synthesis of DPD, which could be used to make both the unnatural (*R*)-DPD, as well as natural (*S*)-DPD (Scheme 8). [77] Their synthesis started from inexpensive d-mannitol **51**, which was protected with an acetal group and then cleaved with NaIO_4_ to provide aldehyde **53** in 43% yield over two steps. Wittig olefination of **53** gave alkene **54** as a mixture of E and Z olefins (70% yield). The lack of control of the alkene geometry was inconsequential because dihydroxylation of both alkenes afforded diastereoisomeric diols **55**, and the mixture was subjected to PCC oxidation to converge to diketone **56**. Deprotection of compound 56 with acid then afforded (*R*)-DPD in a 6.3% overall yield. Natural (*S*)-DPD could be obtained *via* the same route, using the enantiomer of **53**. It has been observed that the absolute configuration at C4 of AI-2 is important for biological activity; in both *E. coli* LsrR-mediated β-gal assay and *V. harveyi* bioluminescence assays, the natural (*S*)*-*DPD was more potent than the unnatural (*R*)-DPD [72,77,78].

**Scheme 9:**
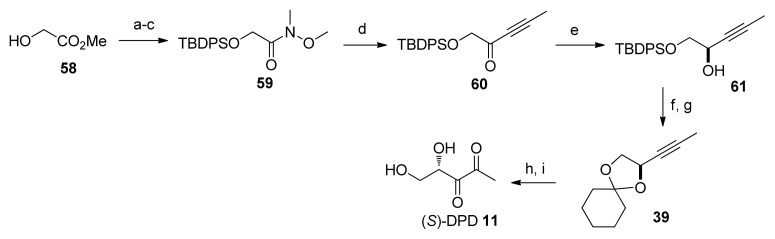
Synthesis of *S*-DPD by Maycock and Ventura. (**a**) TBDPSCl, Pyr, DMAP, rt, 97%; (**b**) LiOH, THF/H_2_O, rt, 94%; (**c**) HNMeOMe·Cl, DCC, CH_2_Cl_2_, rt, Δ, 85%; (**d**) BuLi, propyne, THF, −78 °C/0 °C, 95%; (**e**) (*S*)-Alpine borane, THF, rt, 67%; (**f**) TBAF, THF, rt, 86%; (**g**) 1,1-dimethoxycyclohexanone, H_2_SO_4_, DMF, 91%; (**h**) NaIO_4_, RuO_2_, CCl_4_/MeCN, rt, 86%; (**i**) Dowex 50WX8, H_2_O, pH 3, rt.

Maycock and co-workers have also reported a synthesis of (*S*)-DPD but unlike most enantioselective DPD syntheses that relied on using chiral starting materials, their synthesis used an enantioselective reduction of unsaturated ketone **60** as a key step (Scheme 9) [78]. Hydroxy ester **58** was protected with *tert*-butyldiphenylsilyl group and transformed into Weinreb amide **59** (78% over three steps). Then, the acetylenic group was incorporated via a reaction between the Weinreb amide **59** and lithiated propyne to give **60**. Treatment of **60** with (*S*)-Alpine borane yielded (*R*)-**61** with 86% ee (>98% ee after recrystalization). Then, the silyl protecting group was removed and the diol product was reprotected with cyclohexylidene group to get **39**. Conversion of 39 into DPD followed protocols developed by Semmelhack and Vanderleyen to give (*S*)-DPD in an overall yield of 41%. Of note, compound **39** was first made by Semmelhack and co-workers, so this synthesis of DPD (which entails nine steps, Semmelhack’s synthesis involved seven steps from l-gulonic acid γ-lactone) can be considered as a formal synthesis. With so many different and complementary AI-2 syntheses developed over the years, it should now be possible to make various AI-2 analogs for biological testing.

## 3. AI-2 Signaling Pathway

### 3.1. AI-2 Mediated QS Circuit in *V. harveyi* and *V. cholerae*

**Figure 4:**
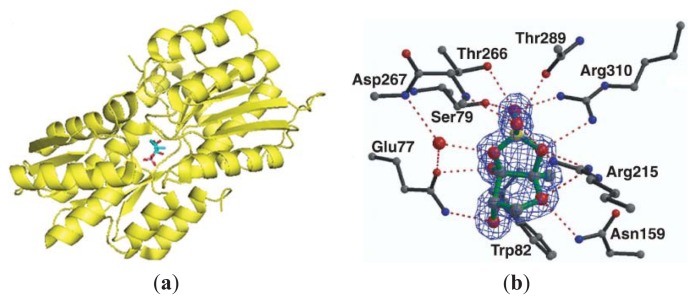
(**a**) LuxP and *S*-THMF-borate in *V. harveyi*. PDB code: 1JX6; (**b**) LuxP binding site. (Adapted from [15] with permission. Copyright 2002 Nature publishing group).

**Figure 5:**
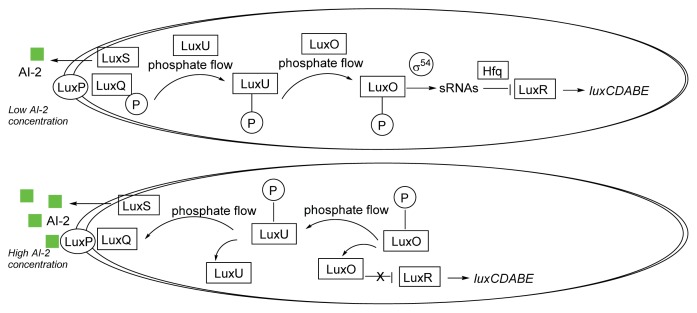
*V. harveyi* AI-2-mediated QS circuit.

AI-2 mediated QS is well studied in the vibrios [79]. In *V. harveyi*, AI-2 is synthesized by LuxS and exported outside, where it binds to an extracellular receptor LuxP (Figure 4), which associates with LuxQ to form LuxPQ to regulate phosphorylation signal transduction cascade (Figure 5) [80]. At low cell density, which correlates with low AI-2 concentration, LuxPQ acts as a kinase and transfers phosphate to LuxU, which then relays a phosphate group to LuxO. LuxO-phosphate (a transcriptional activator), along with sigma factor σ^54^, activates the expression of regulatory small RNAs (sRNAs) Qrr1-5 [81]. Qrr1-5, in conjunction with the chaperone Hfq, destabilizes the *luxR* mRNA so that LuxR synthesis is suppressed. At high AI-2 concentration, AI-2 binds to the LuxPQ complex and the AI-2/LuxPQ complex (Figure 5) converts to a phosphatase, which dephosphorylate LuxU, which in turn also dephosphorylate LuxO. Dephosphorylated LuxO is no longer active and therefore, the concentrations of Qrr1-5, which degrade the mRNA of LuxR, decrease. As the concentration of LuxR, which is a transcription factor, increases, the genes that are controlled by LuxR (some of which are virulence determinants) are expressed. In *V. cholerae*, AI-2 signaling is also mediated by LuxPQ receptor, LuxO and Qrr1-4. sRNAs Qrr1-4 facilitate the degradation of *hapR* mRNA transcript and stabilize *aphA* mRNA transcript. [81] It is worth noting that in both *V. harveyi* and *V. cholerae*, sRNAs Qrr1-5 are not only regulated by AI-2 but also by CAI-1 via the CqsS receptor [19].

### 3.2. AI-2 Mediated QS Circuit in *E. coli* or *S. typhimurium*

**Figure 6:**
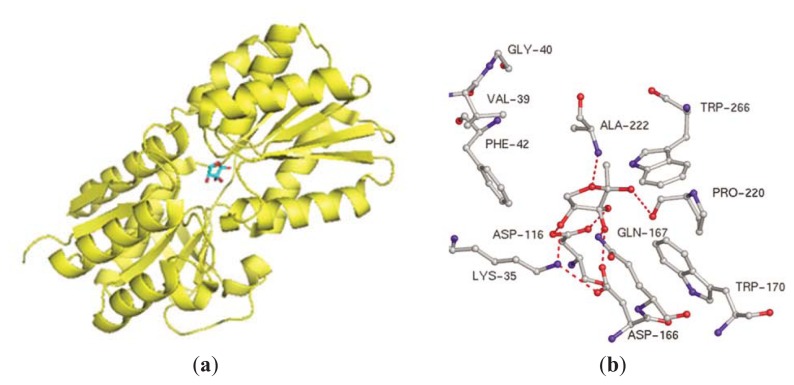
(**a**) LsrB and *R*-THMF in *S. typhimurium.* PDB code: 1TJY; (**b**) LsrB binding site. (Adapted from [84] with permission. Copyright 2004, Elsevier).

**Figure 7:**
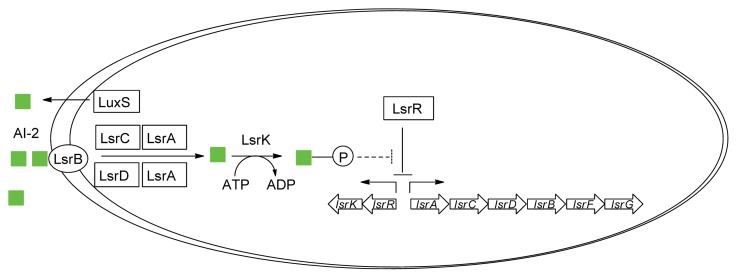
AI-2-mediated QS circuit in *S. typhimurium* and *E. coli*.

In *E. coli*, YdgG has been proposed as a potential AI-2 exporter [82] but as a YdgG mutant still exports AI-2, it is likely that other exporters exist. During bacterial growth, the extracellular concentration of AI-2 increases and when a threshold concentration is reached, AI-2 is transported into the cell via a transporter protein. In the early 2000s, LsrB (periplasmic protein and part of the Lsr ABCD transporter in *S. typhimurium* and *E. coli* responsible for AI-2 internalization) was structurally characterized (Figure 6) [83–85]. Once AI-2 is internalized via LsrB, it is phosphorylated by a kinase, LsrK, and the phospho-AI-2 then binds to the repressor LsrR to de-repress the *lsr* operon (Figure 7) [86]. Thus, at low cell density, when AI-2 concentration is low and hence, the concentration of phospho-AI-2 is also low, LsrR binds the *lsr* promoter to inhibit the transcription or *lsr* genes whereas at high AI-2 concentration, *lsr* genes are transcribed due to the de-repression of LsrR. Both LsrK and LsrR play key regulatory roles in the biofilm formation of *E. coli* [87–89]. It has been shown that the deletion of *lsrR* affects the expression of 146 genes whereas deleting *lsrK* affected 149 genes [87]. *lsrK* or *lsrR* mutants form less biofilms and so small molecules that target these proteins could have anti-biofilm properties.

### 3.3. Other Possible AI-2 Receptors

AI-2 is termed the “universal” autoinducer, yet there is a paucity of identified AI-2 binding receptors. With the exception of LuxPQ and LsrB, both of which have been structurally characterized with a bound ligand and LsrR which has been experimentally characterized as the apo-structure [90], putative AI-2 receptors that presumably sense AI-2 in the myriads of bacteria, which have been shown to respond to AI-2 remain to be identified and structurally characterized. RbsB, a ribose binding protein in *A. actinomycetermcomitans,* has been postulated to be an AI-2 transporter [45]. Xavier and co-workers have also suggested the presence of LsrB-like proteins in *B. anthracis* and *B. cereus* [91] but beyond these putative AI-2 transporters, there has been little success in identifying proteins that respond to AI-2. The discovery of more AI-2 receptors is critical for the development of anti-QS agents that target AI-2 signaling.

## 4. Small Molecule Inhibitors of AI-2 Signaling

### 4.1. AI-2 Synthase Inhibitors

**Figure 8:**
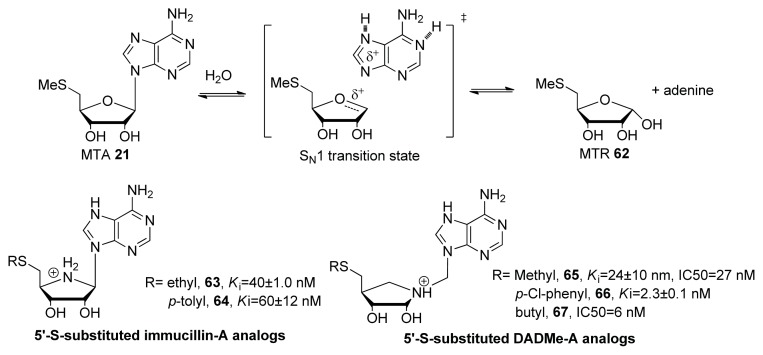
Proposed transition state of MTAN-catalyzed hydrolysis of MTA (top) and potent MTA analogs (bottom) with their inhibition constants (IC_50_) for *S. pneumoniae* (*K*_i_) and *V. cholera* MTAN.

One way to interrupt QS is to inhibit the synthases that produce autoinducers. As shown in Scheme 1, the key enzymes involved in AI-2 biosynthesis are MTAN (5′-methylthioadenosine/*S*-adenosylhomo-cycteine nucleosidase) and LuxS; hence, the inhibition of any of these enzymes would decrease the amount of AI-2. In 1976, it was demonstrated in an important paper that MTA analogs could inhibit MTAN from *E. coli* [92]. Following this discovery, Schramm and co-workers demonstrated that transition state analogs of MTA hydrolysis (Figure 8) strongly inhibited MTAN from several bacteria, including *S. pneumoniae*, *E. coli* and *V. cholerae* [93,94]. 5′-*S*-substituted immucillin-A analogs aim to mimic an early transition state where ribosyl and adenine bond is partially broken while 5′-*S*-substituted immucillin DADMe analogs mimic a late transition state whereby adenine is fully dissociated (Figure 8). MTA is also a substrate for the human MTA phosphorylase hence it is possible that some MTA analogs could inhibit the human enzyme to cause toxicity. There are however structural differences between the bacterial MTA nucleosidase and the human MTA phosphorylase to allow for selective targeting of the bacterial enzyme [95,96].

**Figure 9:**
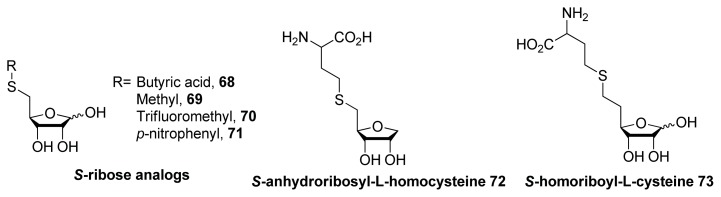
SRH analogs prepared by Zhou and co-workers [97,98].

**Figure 10:**
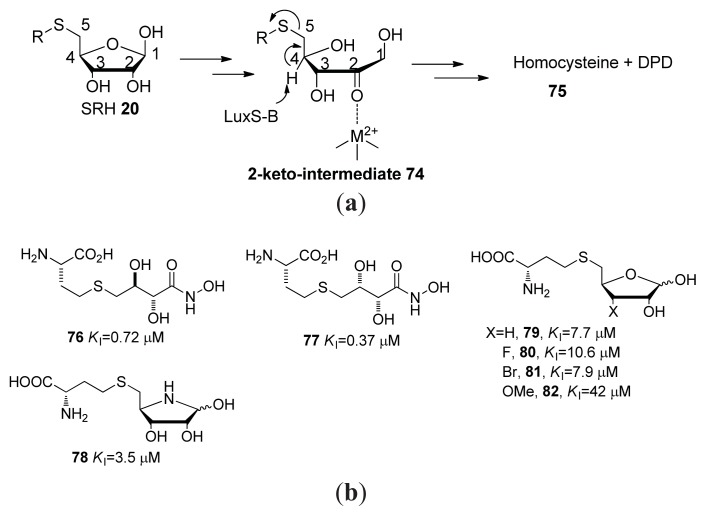
(**a**) Proposed mechanism of LuxS catalyzed cleavage of SRH; (**b**) SRH or cleavage intermediate analogs, with their inhibition constants to LuxS from *B. subtilis*.

LuxS catalyzes the conversion of SRH into AI-2 and hence, the inhibition of LuxS is a viable strategy to reduce the concentration of AI-2. Zhou and co-workers designed and synthesized SRH analogs to inhibit LuxS (Figure 9) [97,98]. Pei and co-workers proposed a LuxS catalyzed SRH cleavage mechanism (Figure 10a) [99] and to mimic the 2-keto-intermediate **74** and SRH, they developed analogs shown in Figure 10b [100–103]. Some of the analogs developed by Pei and co-workers showed a good inhibition profile (submicromolar *K*_i_) against LuxS.

**Figure 11:**
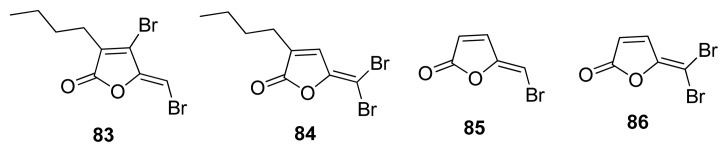
Structures of brominated furanone LuxS inhibitors.

**Scheme 10:**
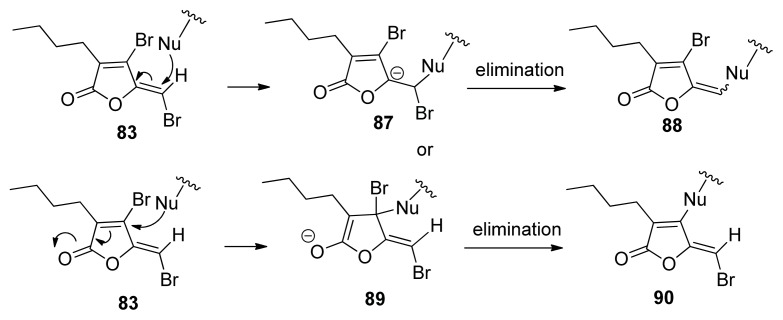
Two proposed inhibition pathways of LuxS by brominated furanones. A nucleophile in LuxS residues either adds to the exocyclic vinyl or ring vinyl bond. After elimination of bromide, both mechanisms increase LuxS mass by about 229 Da. (Adapted from [55] with permission. Copyright 2009, Elsevier).

A series of naturally occurring brominated furanones were isolated from the red marine alga *Delisa pulchra* by Gram, Givskov *et al.* in 1996 [104,105]. These have been shown to be potent anti-biofilm and anti-QS inhibitors [106,107]. Zhou and co-workers have shown that brominated furanones, such as **83**–**86** (Figure 11), are LuxS covalent inhibitors of LuxS (see Scheme 10 for proposed mechanism of inhibition) [55]. Brominated furanones do not only inhibit LuxS but also other proteins involved in AI-1 perception [108]. A recent study showed that analogs of brominated furanones, with attenuated toxicity, have interesting anti-biofilm properties against *E. coli* and *P. aeruginosa* [109].

Han and Lu reported in 2009 that LuxS could also be inhibited with the peptide, TNRHNPHHLHHV [110]. More work is needed to reveal if peptidase-resistant analogs of this promising peptide could be used to quench AI-2 signaling *in vivo*.

Apart from the inhibition of AI-2 synthesis, others have also suggested the sequestration or modification of AI-2 as a viable means to quench AI-2 signaling. For example, Bentley and co-workers demonstrated a proof-of-concept modification of AI-2 in culture media, using the kinase LsrK and ATP [111]. The phosphorylated AI-2 was then unable to cross into the bacterial cytosol and hence, AI-2 signaling was quenched. Alexander and co-workers have also sequestered AI-2 from bacterial culture media by using polymeric material that contains boron, which chelates AI-2 [112].

### 4.2. AI-2 Receptor QS Inhibitors

**Figure 12:**
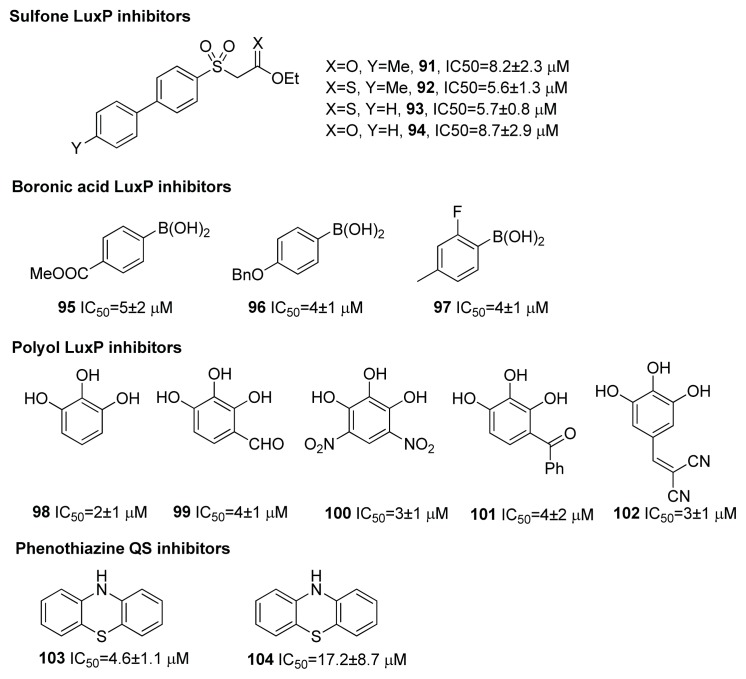
LuxP inhibitors in *V. harveyi*.

Utilizing high-throughput virtual screening on *V. harveyi* LuxP crystal structure, Wang and co-workers found a few sulfone compounds, from 1.7 million commercially available or easy-to-synthesize molecules, which could antagonize QS in *V. harveyi* (Figure 12) [113,114]. They proposed that the sulfone group is critical to the activity because it interacts with Arg215 and Arg310 of LuxP, similar to the binding of *S*-THMF-borate.

**Figure 13:**
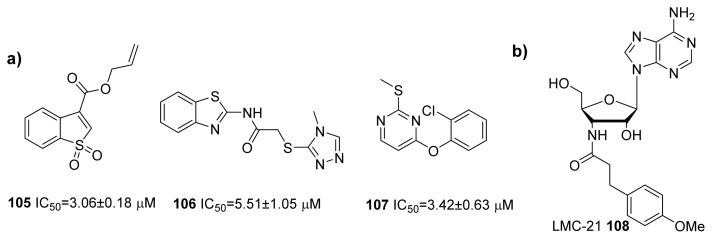
LuxPQ inhibitors tested by *V. harveyi*. (**a**) LuxPQ inhibitors, identified by Wang and co-workers [119]; (**b**) LMC-21, developed by Coenye and co-workers [120].

The same group also envisioned that boronic acids and polyol structures could mimic *S*-THMF-borate, which binds to LuxP, and found a series of *p*-substituted phenylboronic acids **95**–**97** [115,116] and aromatic polyols **98**–**102**, which antagonize QS in *V. harveyi* (Figure 12) [117]. They have also reported that phenothiazine **103** and **104** (Figure 12) inhibit both AI-2 and AI-1 based QS in *V. harveyi*, although the mechanism of inhibition by these compounds is currently not understood [118]. Continuing in the same theme of using virtual screening to identify new inhibitors of AI-2 signaling, Wang and co-workers identified compounds **105**–**107** (Figure 13a) as inhibitors of LuxPQ complex [119].

Others have also identified interesting compound classes that inhibit AI-2 signaling in bacteria. Coenye and co-workers initially evaluated nucleoside analogs hoping that some adenosine analogs could inhibit AI-2 synthase, due to its similarity with SAH, which is a substrate for LuxS. Surprisingly, they found LMC-21, **108** (which contains some of the SAH motifs, see Figure 13b) as an inhibitor of AI-2 signaling through potential binding to LuxPQ, and not necessarily via LuxS inhibition, in *V. harveyi* [120].

**Figure 14:**
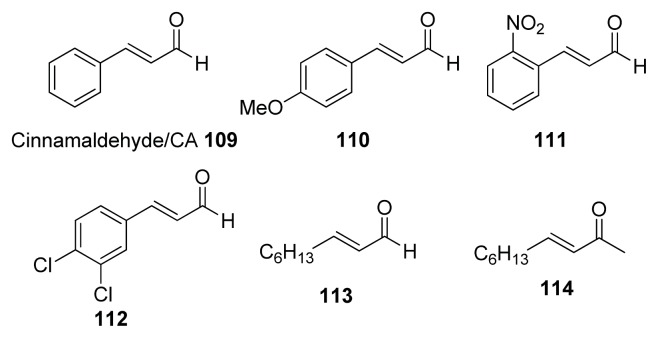
Structures of cinnamaldehyde and analogs.

Coenye and co-workers, as well as Gilbert *et al.*, have shown that cinnamaldehyde has anti-QS activity. Cinnamaldehyde **109** and analogs **110**–**114** (Figure 14) have been found to inhibit AI-2 signaling in *Vibrio* spp. and it is believed the target protein is LuxR [121–123].

**Figure 15:**
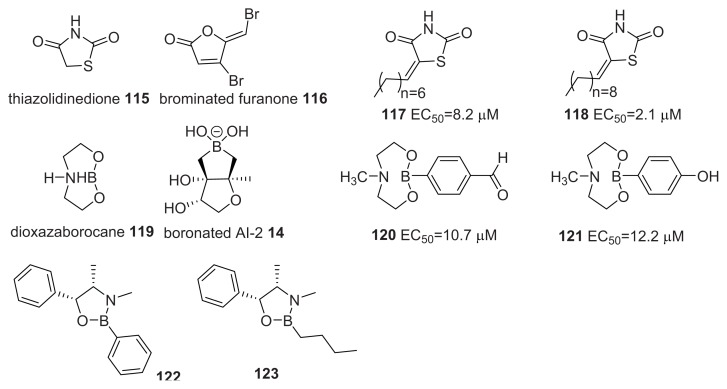
Thiazolidinediones and dioxazaborocanes synthesized by Brackman *et al.* [124] and tested as QS inhibitors in *V. harveyi*.

Brackman and co-workers designed and evaluated two libraries of compounds (thiazolidinediones and dioxazaborocanes) for anti-QS activities. The authors suggested that the thiazolidinediones (such as **115**, **117** and **118**, see Figure 15) are structural mimics of brominated furanones, such as **117** and **118**, and hence, could potentially act on biomolecular targets that furanones are known to target, such as LuxS. Also, the dioxazaborocanes (**119**–**123**) resemble boronated AI-2 (see Figure 15) [124,125]. A few of them (**119**–**121**) acted as potent AI-2 QS inhibitors against *V. harveyi*, with *EC*_50_ at low micromolar level (see Figure 15 for *EC*_50_ values).

**Figure 16:**
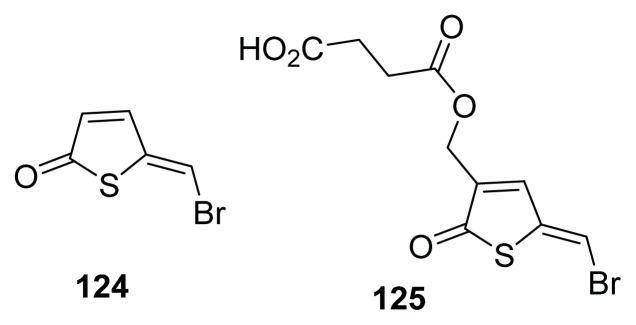
Brominated thiophenone QS inhibitors.

Defoirdt and co-workers made the sulphur analogs of brominated furanones (brominated thiophenones, such as **124** and **125** (Figure 16), and showed that at low micromolar concentrations these compounds could block quorum sensing in *V. harveyi*, via the QS master regulator LuxR [126].

### 4.3. AI-2 Analogs as QS Inhibitors

**Figure 17:**
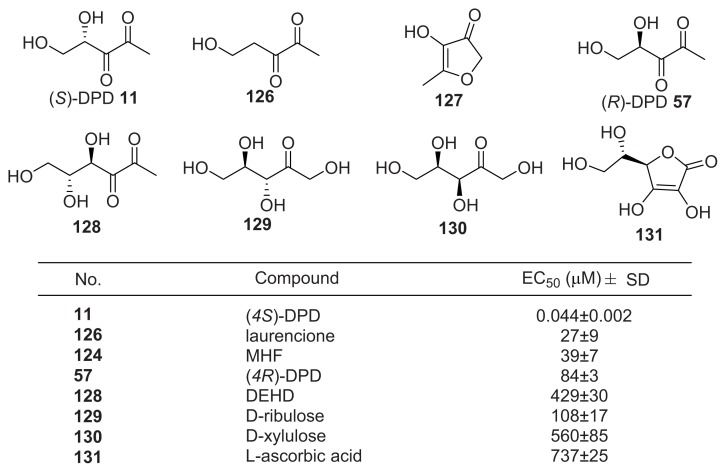
Probing specificity of LuxP binding site with AI-2-like molecules. (Adapted from [127] with permission. Copyright 2005, Elsevier).

A simple trick in discovering antagonists of a natural ligand is to modify that ligand. Several groups have therefore modified AI-2, with the hope of arriving at analogs that could interfere with AI-2 signaling. Janda and co-workers tested a panel of AI-2-like molecules (see Figure 17), using bioluminescence of *V. harveyi* as read out and concluded that the oxidation states at C2, C3 and C4 were important for the biological activity of AI-2 with *V. harveyi*. Also, the absolute configuration at C4 was critical for activity as the natural *S*-enantiomer had an *EC*_50_ of 0.044 μM whereas the *R*-enentiomer had an *EC*_50_ of 84 μM (almost 2000-fold reduction) [127].

**Figure 18:**
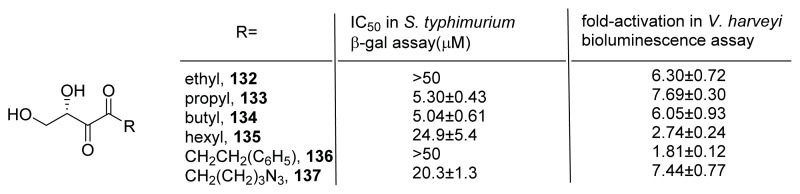
Janda’s C1 substituted DPD analogs [62]. IC_50_ in *S. typhimurium* is for the inhibition of AI-2-promoted expression of β-galactosidase (indirectly measured via β-gal enzymatic assay) whereas the fold-activation in *V. harveyi* signifies the potentiation of AI-2 agonism by analogs, which are not agonists on their own.

**Figure 19:**
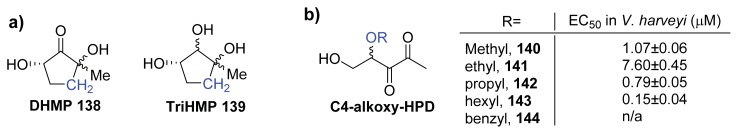
(**a**) Carbocyclic and (**b**) C4-alkoxy AI-2 analogs developed in Janda’s laboratory [63,64].

Janda and co-workers also synthesized a panel of C1-substituted alkyl-DPD analogs (Figure 18) [62]. These analogs were evaluated in *V. harveyi* MM32 and *S. typhimurium* using bioluminescence assay and β-gal assay, respectively. Synergistic agonist activity (that is, the analogs did not have any agonist activity on their own but potentiated the activity of AI-2) was found in *V. harveyi* MM32 whereas antagonist activity was found, especially for propyl-**133** and butyl-DPD **134**, with *IC*_50_ of around 5 μM in the presence of 50 μM AI-2 in *S. typhimurium*. The same group has also made carbocyclic AI-2 analogs **138** and **139** (Figure 19a) as well as replacing the hydroxyl group at C4 of AI-2 with alkoxy groups **140**–**144** (Figure 19b) [63,64]. The carbocyclic analogs did not display significant QS quenching activities in *S. typhimurium* or *V. harveyi*, whereas the C4 alkoxy analogs of DPD showed potent agonist activity in *V. harveyi* (see Figure 19b).

*P. aeruginosa* does not make AI-2 but can sense AI-2 produced by other bacteria. Meijler and co-workers tested AI-2 analogs on *P. aeruginosa* and found that these had anti-QS activities against this bacterium [70].

**Figure 20:**
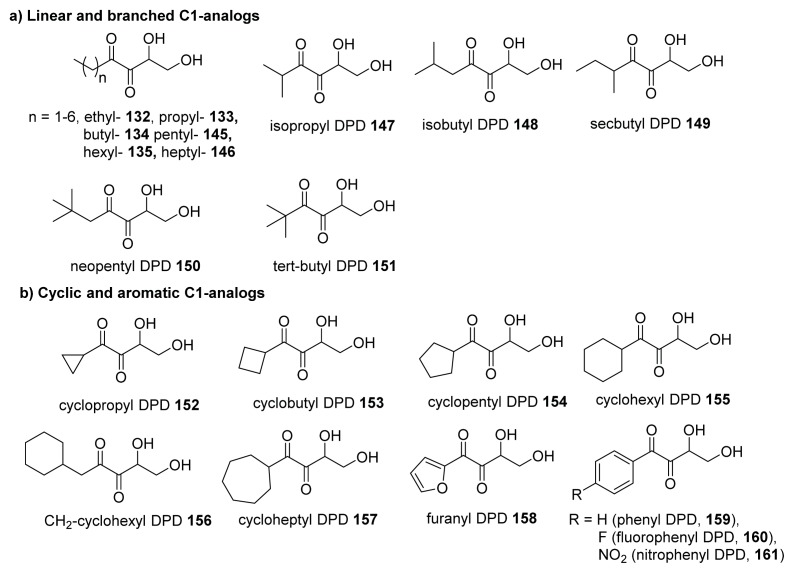
C1 substituted AI-2 analogs synthesized and evaluated by Sintim and co-workers. (**a**) Linear and branched C1-analogs; (**b**) Cyclic and aromatic C1-analogs. (Taken from [67] with permission. Copyright 2012, American Chemical Society).

**Figure 21:**
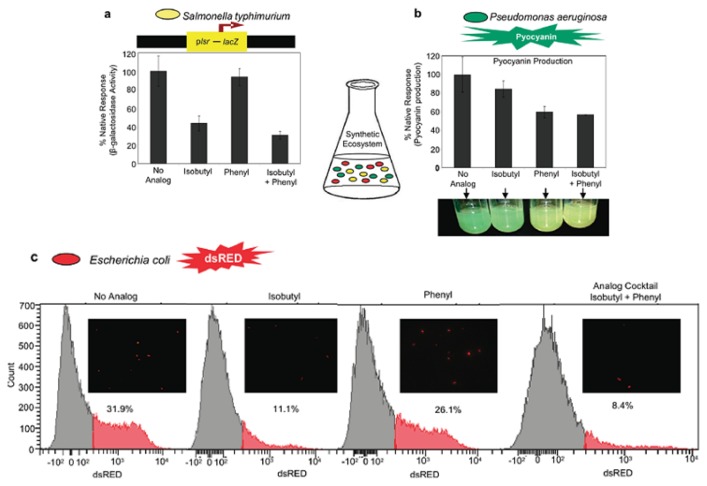
Effect of alkyl AI-2 analogs and analog cocktail in a trispecies synthetic ecosystem. (**a**) AI-2 dependent β-gal assay in *S. typhimurium* MET708; (**b**) QS related pyocyanin production in *P. aeruginosa* PAO1; (**c**) AI-2 dependent dsRED induction in *E. coli* W3110 pCT6 dsRED, in response of 40 μM analogs individually and a cocktail of both. (Adapted from [67] with permission. Copyright 2012, American Chemical Society).

Sintim’s synthesis of AI-2, see Scheme 7, which utilized diazocarbonyls, is modular and hence, has facilitated the synthesis of a diverse C1 alkyl library of AI-2 analogs for further biological evaluation (Figure 20). Sintim and co-workers first made various C1-akyl DPD analogs, including isopropyl-, *t*-butyl-, cyclopropyl- and cyclohexyl analogs to investigate the effect of different alkyl chain sizes and shapes in binding to *V. harveyi* [65]. They also observed synergistic agonism of AI-2 analogs in the presence of AI-2 and hypothesized that AI-2 receptors that were responsible for the synergistic agonism were promiscuous [65]. Subsequent works from the Sintim laboratory expanded on the diversification of the C1 side chain of AI-2 by making C1 linear, branched, cyclic alkyl as well as C1 aromatic AI-2 analogs (Figure 20). It was discovered that, unlike AI-2 that mainly enters enteric bacteria via the Lsr transporters, C1 alkyl AI-2 analogs can freely diffuse into bacterial cells [128] and that isobutyl DPD was a potent QS inhibitor in both *E. coli* and *S. typhimurium* [66] and probably acted via LsrR after phosphorylation by LsrK. Of note, AI-2 analogs still maintained their inhibitory activities in polymicrobial systems (Figure 21), which mimic natural ecosystems better than monocultures that have traditionally been used in anti-QS assays. The facile preparation of diverse AI-2 analogs allowed for investigations into the specificities of AI-2 for LsrK and LsrR. Sintim, Bentley and co-workers discovered that both LsrK and LsrR are promiscuous and that most C1-modified analogs of AI-2 could be phosphorylated by LsrK [66,67]. Recently Janda and co-workers have used a more detailed kinetic analysis of LsrK phosphorylation of AI-2 analogs to confirm the initial findings by Sintim and co-workers [129]. Just like LsrK, LsrR is also promiscuous and binds to several phosphorylated AI-2 analogs. There are some nuances with the binding of phosphorylated AI-2 analogs to LsrR and whereas C1 methyl and ethyl analogs of phospho-AI-2 bind to LsrR to de-repress this repressor from DNA to allow for *lsr* expression, C1 propyl and higher alkyls bind to LsrR to increase the repression of *lsr* expression [66].

**Figure 22:**
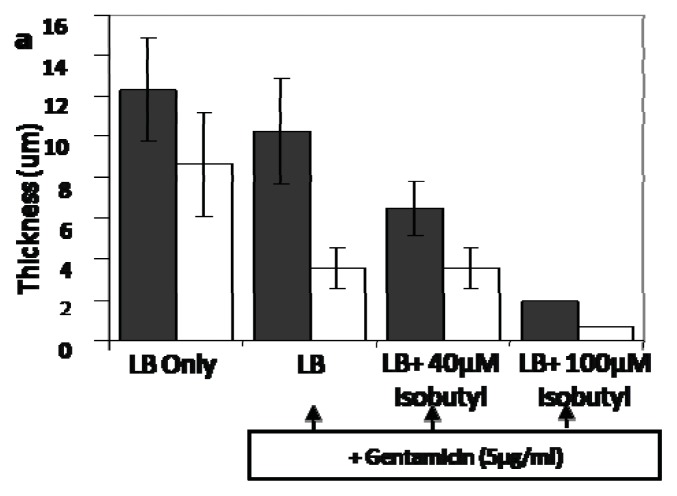

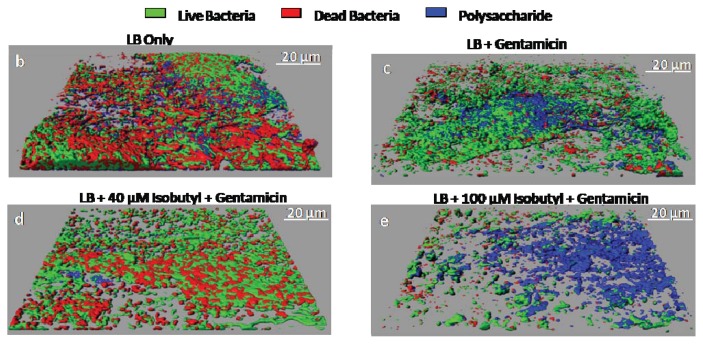
Effect of combinatorial approach of analog and gentamicin on performed E. coli biofilm thickness and architecture. (**a**) thickness and biomass of biofilm analyzed by COMSTAT; (**b**)–(**e**) representative Imaris 3D surface reconstructions of the biofilm with (**b**) LB only; (**c**) LB+5 μg/mL Gentamicin; (**d**) LB+40 μM isobutyl-DPD 148+5μg/mL gentamicin; (**e**) LB+100 μM isobutyl-DPD 148 +5μg/mL gentamicin. (Adapted from ref. [44] with permission. Copyright 2013, Springer).

Bacterial biofilms are notoriously difficult to treat and are resistant to many antibiotics [130–136]. Recently, it was demonstrated that isobutyl DPD, in combination with the antibiotic gentamicin, could almost completely clear pre-existing *E. coli* biofilms (Figure 22) [31]. This adds to an emerging trend of using anti-QS agents to potentiate the effects of traditional antibiotics [137].

**Figure 23:**
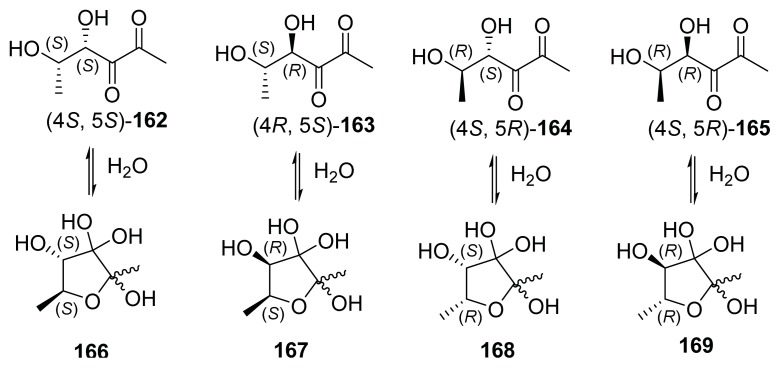
C5 analogs of AI-2 developed by Ventura and co-workers. These analogs contain stereochemical diversity at the C4 and C5 positions. (Adapted from [71] with permission. Copyright 2012, Elsevier).

Most AI-2 analogs reported in the last few years have focused on C1 modification [62,67,70]. Recently, Ventura and co-workers showed that C5-modified AI-2 analogs were synergistic agonists in *E. coli* and strong agonists in *V. harveyi* (Figure 23) [71].

**Figure 24:**
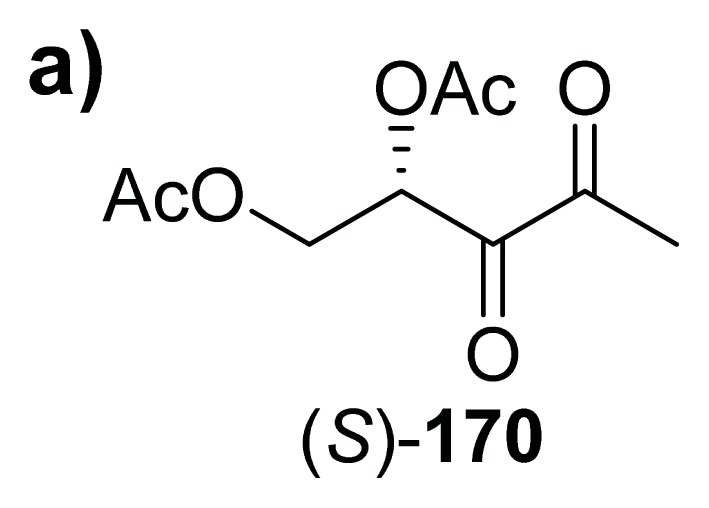

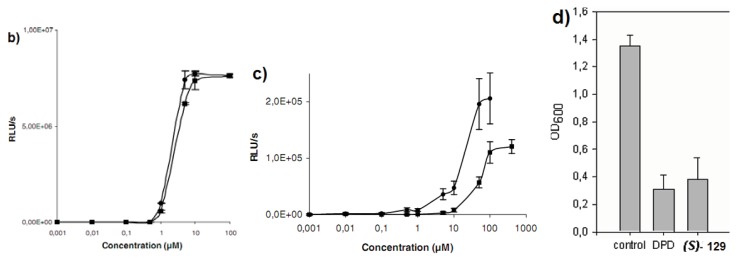
Acetate-protected AI-2 synthesized by Doutheau and co-workers. (**a**) Structure of bis-(*O*)-acetylated-DPD; (**b**) Bioluminescence induction in *V. harveyi*, (*S*)-**170** (●) and (*S*)-DPD **11**(■); (**c**) β-gal production in *S. typhimurium*, (*S*)-**170** (●) and (*S*)-DPD **11**(■); (**d**) Biofilm inhibition in *B. cereus*, (*S*)-**170** (●) and (*S*)-DPD **11** (■) at 8 μM; (Adapted from [138] with permission. Copyright 2007, Elsevier).

Both AI-2 and analogs have issues with stability and to address the potential instability of AI-2 analogs [61], Doutheau and co-workers demonstrated that acetate protected analogs of AI-2 were as effective as natural AI-2 but had the added advantage of being stable (see Figure 24).

**Figure 25:**
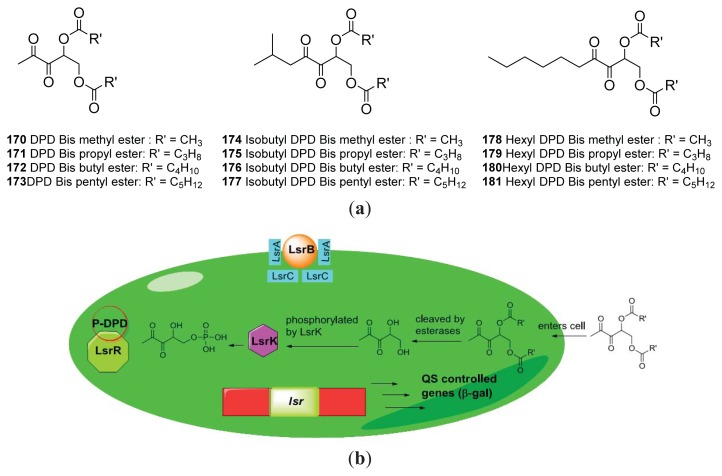
Bis-ester protected AI-2 analogs and proposed model of action in enteric bacteria. (**a**) Sructures of *bis*-ester protected AI-2 analogs; (**b**) Proposed pro-drug activation and processing of AI-2 in enteric bacteria. Ester protected AI-2 analogs diffuse into the bacterial cell. The analogs are cleaved by esterase and are subsequently phosphorylated by LsrK. (Taken from [68] with permission. Copyright 2012, MDPI).

Following Doutheau’s work, Sintim and co-workers asked if ester-protected analogs of AI-2 could also be hydrolyzed in bacterial cells to reveal active antagonists (Figure 25) [68,138]. For this work, Sintim and co-workers screened the effect of the alkyl chain of the esters used to protect AI-2 analogs. Ester-protected AI-2 analogs could also be hydrolyzed by endogenous bacterial esterases and showed potent anti-QS profiles that were similar to the unprotected AI-2 analogs [68].

### 4.4. Inhibition of AI-2 QS by Dietary Compounds

**Figure 26:**
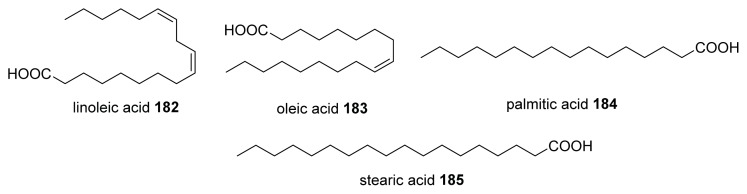
Fatty acid AI-2 inhibitors.

Pillai and co-workers focused on investigating the influence of some food matrices on AI-2 mediated QS. They identified certain fatty acids, which inhibited *V. harveyi* AI-2 activity at micro molar levels (Figure 26) [139,140].

## 5. Conclusions

AI-2 as a universal signaling molecule in bacteria has had a “chequered” history. First hailed as an almost ubiquitous signaling molecule in bacteria, it has also been described as a metabolic by-product, and not a quorum sensing molecule, in bacteria [50,141,142]. AI-2’s claim to being a bona fide QS autoinducer has not been helped by the lack of identified cellular receptors in bacteria. So far, only a handful of receptors have been found to bind to AI-2. Whether AI-2 is a signal or a cue, by virtue of it being a metabolic waste [143], an increasing number of bacterial behaviors have now been shown to be regulated by the actual AI-2 molecule or by LuxS, which synthesizes AI-2 or both [39,53,144]. Small molecules that target LuxS or known receptors of AI-2 (such as LsrR) or the yet-to-be identified receptors that bind to AI-2 will certainly have some utility in modulating bacterial behavior and might even have clinical applications as anti-biofilm agents [31]. Recently, there has been a surging interest in making “biobricks” for synthetic biology applications. Quorum sensing receptors are prime building blocks for making engineered bacterial cells and antagonists of AI-2 signaling could serve as modulators of AI-2-based synthetic circuits.

## Figures and Tables

**Table 1:** Some bacterial virulence determinants, which are regulated by AI-2/LuxS.

Organism	Signaling molecules	Receptors	Phenotype
*E. coli*	AI-2	LsrB, LsrR	Motility [33], biofilm formation [34]
*S. typhimurium*	AI-2	LsrB, LsrR	InvF [35]
*S. aeurus*	AI-2, AIP	AgrC, AgrA	Biofilm formation [36]
*S. anginosus*	AI-2	unknown	Susceptibility to antibiotics [37]
*M. catarrhalis*	AI-2	unknown	Biofilm formation and antibiotic resistance [38]
*H. pylori*	AI-2	TlpB	Motility [39]
*V. cholerae*	CAI-1, AI-2	CqsS, LuxP	Biofilm formation, virulence factor production and protease [19,40]
*V. harveyi*	HAI-1, CAI-1, AI-2	LuxN, CqsS, LuxP	Bioluminescence, biofilm formation, colony morphology, siderophore production, type III secretion and metalloprotease production [12,41,42]
*V. fischeri*	3-oxo-C_6_-HSL, C_8_-HSL, AI-2	AinR, LuxP, LuxR	Bioluminescence [43]
*Y. pestis*	3-oxo-C_8_-HSL, 3-oxo-C_6_-HSL, AI-2	LuxR homologue	Virulence factor expression [44]
*A. actinomycetemcomitans*	AI-2	RbsB, LsrB	Optimal growth under iron starvation and biofilm development [45]

## References

[1] Mullard A. (2011). 2010 FDA drug approvals. Nat. Rev. Drug Discov.

[2] Mullard A. (2012). 2011 FDA drug approvals. Nat. Rev. Drug Discov.

[3] Mullard A. (2013). 2012 FDA drug approvals. Nat. Rev. Drug Discov.

[4] Sintim H.O., Smith J.A., Wang J., Nakayama S., Yan L. (2010). Paradigm shift in discovering next-generation anti-infective agents: Targeting quorum sensing, c-di-GMP signaling and biofilm formation in bacteria with small molecules. Future Med. Chem.

[5] Bassler B.L. (1999). How bacteria talk to each other: Regulation of gene expression by quorum sensing. Curr. Opin. Microbiol.

[6] Antunes L.C., Ferreira R.B., Buckner M.M., Finlay B.B. (2010). Quorum sensing in bacterial virulence. Microbiology.

[7] Fuqua W.C., Winans S.C., Greenberg E.P. (1994). Quorum sensing in bacteria: The LuxR-LuxI family of cell density-responsive transcriptional regulators. J. Bacteriol.

[8] Tomasz A. (1965). Control of the competent state in *Pneumococcus* by a hormone-like cell product: An example for a new type of regulatory mechanism in bacteria. Nature.

[9] Lazazzera B.A., Grossman A.D. (1998). The ins and outs of peptide signaling. Trends Microbiol.

[10] Nealson K.H., Platt T., Hastings J.W. (1970). Cellular control of the synthesis and activity of the bacterial luminescent system. J. Bacteriol.

[11] Nealson K.H. (1977). Autoinduction of bacterial luciferase. Occurrence, mechanism and significance. Arch. Microbiol.

[12] Henke J.M., Bassler B.L. (2004). Quorum sensing regulates type III secretion in *Vibrio harveyi* and *Vibrio parahaemolyticus*. J. Bacteriol.

[13] Eberhard A., Burlingame A.L., Eberhard C., Kenyon G.L., Nealson K.H., Oppenheimer N.J. (1981). Structural identification of autoinducer of *Photobacterium fischeri* luciferase. Biochemistry.

[14] Magnuson R., Solomon J., Grossman A.D. (1994). Biochemical and genetic characterization of a competence pheromone from *B. subtilis*. Cell.

[15] Chen X., Schauder S., Potier N., Van Dorsselaer A., Pelczer I., Bassler B.L., Hughson F.M. (2002). Structural identification of a bacterial quorum-sensing signal containing boron. Nature.

[16] Pesci E.C., Milbank J.B., Pearson J.P., McKnight S., Kende A.S., Greenberg E.P., Iglewski B.H. (1999). Quinolone signaling in the cell-to-cell communication system of *Pseudomonas aeruginosa*. Proc. Natl. Acad. Sci. USA.

[17] Takano E. (2006). Gamma-butyrolactones: *Streptomyces* signalling molecules regulating antibiotic production and differentiation. Curr. Opin. Microbiol.

[18] Khokhlov A.S., Tovarova I.I., Borisova L.N., Pliner S.A., Shevchenko L.N., Kornitskaia E., Ivkina N.S., Rapoport I.A. (1967). The A-factor, responsible for streptomycin biosynthesis by mutant strains of *Actinomyces streptomycini*. Dokl. Akad. Nauk SSSR.

[19] Miller M.B., Skorupski K., Lenz D.H., Taylor R.K., Bassler B.L. (2002). Parallel quorum sensing systems converge to regulate virulence in *Vibrio cholerae*. Cell.

[20] Higgins D.A., Pomianek M.E., Kraml C.M., Taylor R.K., Semmelhack M.F., Bassler B.L. (2007). The major *Vibrio cholerae* autoinducer and its role in virulence factor production. Nature.

[21] Barber C.E., Tang J.L., Feng J.X., Pan M.Q., Wilson T.J., Slater H., Dow J.M., Williams P., Daniels M.J. (1997). A novel regulatory system required for pathogenicity of *Xanthomonas campestris* is mediated by a small diffusible signal molecule. Mol. Microbiol.

[22] Kesarwani M., Hazan R., He J., Que Y.A., Apidianakis Y., Lesic B., Xiao G., Dekimpe V., Milot S., Deziel E. (2011). A quorum sensing regulated small volatile molecule reduces acute virulence and promotes chronic infection phenotypes. PLoS Pathog.

[23] Holden M.T., Ram Chhabra S., de Nys R., Stead P., Bainton N.J., Hill P.J., Manefield M., Kumar N., Labatte M., England D. (1999). Quorum-sensing cross talk: Isolation and chemical characterization of cyclic dipeptides from *Pseudomonas aeruginosa* and other gram-negative bacteria. Mol. Microbiol.

[24] Lee J., Wu J., Deng Y., Wang J., Wang C., Chang C., Dong Y., Williams P., Zhang L.H. (2013). A cell-cell communication signal integrates quorum sensing and stress response. Nat. Chem. Biol.

[25] Havarstein L.S., Coomaraswamy G., Morrison D.A. (1995). An unmodified heptadecapeptide pheromone induces competence for genetic transformation in *Streptococcus pneumoniae*. Proc. Natl. Acad. Sci. USA.

[26] Taga M.E., Bassler B.L. (2003). Chemical communication among bacteria. Proc. Natl. Acad. Sci. USA.

[27] Federle M.J., Bassler B.L. (2003). Interspecies communication in bacteria. J. Clin. Invest.

[28] McKnight S.L., Iglewski B.H., Pesci E.C. (2000). The *Pseudomonas* quinolone signal regulates rhl quorum sensing in *Pseudomonas aeruginosa*. J. Bacteriol.

[29] Kelly R.C., Bolitho M.E., Higgins D.A., Lu W., Ng W.L., Jeffrey P.D., Rabinowitz J.D., Semmelhack M.F., Hughson F.M., Bassler B.L. (2009). The *Vibrio cholerae* quorum-sensing autoinducer CAI-1: Analysis of the biosynthetic enzyme CqsA. Nat. Chem. Biol.

[30] Schauder S., Bassler B.L. (2001). The languages of bacteria. Genes Dev.

[31] Roy V., Meyer M.T., Smith J.A., Gamby S., Sintim H.O., Ghodssi R., Bentley W.E. (2013). AI-2 analogs and antibiotics: A synergistic approach to reduce bacterial biofilms. Appl. Microbiol. Biotechnol.

[32] Pereira C.S., Thompson J.A., Xavier K.B. (2013). AI-2-mediated signalling in bacteria. FEMS Microbiol. Rev.

[33] Sperandio V., Torres A.G., Giron J.A., Kaper J.B. (2001). Quorum sensing is a global regulatory mechanism in enterohemorrhagic *Escherichia coli* O157:H7. J. Bacteriol.

[34] Wood T.K. (2009). Insights on *Escherichia coli* biofilm formation and inhibition from whole-transcriptome profiling. Environ. Microbiol.

[35] Choi J., Shin D., Ryu S. (2007). Implication of quorum sensing in *Salmonella enterica* serovar typhimurium virulence: The *luxS* gene is necessary for expression of genes in pathogenicity island 1. Infect. Immun.

[36] Yarwood J.M., Bartels D.J., Volper E.M., Greenberg E.P. (2004). Quorum sensing in *Staphylococcus aureus* biofilms. J. Bacteriol.

[37] Ahmed N.A., Petersen F.C., Scheie A.A. (2007). AI-2 quorum sensing affects antibiotic susceptibility in *Streptococcus anginosus*. J. Antimicrob. Chemother.

[38] Armbruster C.E., Hong W., Pang B., Weimer K.E., Juneau R.A., Turner J., Swords W.E. (2010). Indirect pathogenicity of *Haemophilus influenzae* and *Moraxella catarrhalis* in polymicrobial otitis media occurs via interspecies quorum signaling. MBio.

[39] Rader B.A., Wreden C., Hicks K.G., Sweeney E.G., Ottemann K.M., Guillemin K. (2011). *Helicobacter pylori* perceives the quorum-sensing molecule AI-2 as a chemorepellent via the chemoreceptor TlpB. Microbiology.

[40] Hammer B.K., Bassler B.L. (2003). Quorum sensing controls biofilm formation in *Vibrio cholerae*. Mol. Microbiol.

[41] Bassler B.L., Wright M., Silverman M.R. (1994). Multiple signalling systems controlling expression of luminescence in *Vibrio harveyi*: Sequence and function of genes encoding a second sensory pathway. Mol. Microbiol.

[42] Waters C.M., Bassler B.L. (2006). The *Vibrio harveyi* quorum-sensing system uses shared regulatory components to discriminate between multiple autoinducers. Genes Dev.

[43] Engebrecht J., Nealson K., Silverman M. (1983). Bacterial bioluminescence: Isolation and genetic analysis of functions from *Vibrio fischeri*. Cell.

[44] Gelhaus H.C., Rozak D.A., Nierman W.C., Chen D., Varga J.J., Zadeh M., Ulrich R.L., Adamovicz J.J. (2009). Exogenous *Yersinia pestis* quorum sensing molecules *N*-octanoyl-homoserine lactone and *N*-(3-oxooctanoyl)-homoserine lactone regulate the LcrV virulence factor. Microb. Pathog.

[45] James D., Shao H., Lamont R.J., Demuth D.R. (2006). The *Actinobacillus actinomycetemcomitans* ribose binding protein RbsB interacts with cognate and heterologous autoinducer 2 signals. Infect. Immun.

[46] Geske G.D., O’Neill J.C., Blackwell H.E. (2008). Expanding dialogues: From natural autoinducers to non-natural analogues that modulate quorum sensing in Gram-negative bacteria. Chem. Soc. Rev.

[47] Galloway W.R., Hodgkinson J.T., Bowden S.D., Welch M., Spring D.R. (2011). Quorum sensing in Gram-negative bacteria: Small-molecule modulation of AHL and AI-2 quorum sensing pathways. Chem. Rev.

[48] LaSarre B., Federle M.J. (2013). Exploiting quorum sensing to confuse bacterial pathogens. Microbiol. Mol. Biol. Rev.

[49] Schauder S., Shokat K., Surette M.G., Bassler B.L. (2001). The LuxS family of bacterial autoinducers: Biosynthesis of a novel quorum-sensing signal molecule. Mol. Microbiol.

[50] Winzer K., Hardie K.R., Burgess N., Doherty N., Kirke D., Holden M.T., Linforth R., Cornell K.A., Taylor A.J., Hill P.J. (2002). LuxS: Its role in central metabolism and the *in vitro* synthesis of 4-hydroxy-5-methyl-3(2*H*)-furanone. Microbiology.

[51] Surette M.G., Miller M.B., Bassler B.L. (1999). Quorum sensing in *Escherichia coli*, *Salmonella typhimurium*, and *Vibrio harveyi*: A new family of genes responsible for autoinducer production. Proc. Natl. Acad. Sci. USA.

[52] Lerat E., Moran N.A. (2004). The evolutionary history of quorum-sensing systems in bacteria. Mol. Biol. Evol.

[53] Hardie K.R., Heürlier K. (2008). Establishing bacterial communities by “word of mouth”: LuxS and autoinducer 2 in biofilm development. Nat. Rev. Microbiol.

[54] Hauck T., Hubner Y., Bruhlmann F., Schwab W. (2003). Alternative pathway for the formation of 4,5-dihydroxy-2,3-pentanedione, the proposed precursor of 4-hydroxy-5-methyl-3(2*H*)-furanone as well as autoinducer-2, and its detection as natural constituent of tomato fruit. Biochim. Biophys. Acta.

[55] Zang T., Lee B.W., Cannon L.M., Ritter K.A., Dai S., Ren D., Wood T.K., Zhou Z.S. (2009). A naturally occurring brominated furanone covalently modifies and inactivates LuxS. Bioorg. Med. Chem. Lett.

[56] Globisch D., Lowery C.A., McCague K.C., Janda K.D. (2012). Uncharacterized 4,5-dihydroxy-2,3-pentanedione (DPD) molecules revealed through NMR spectroscopy: Implications for a greater signaling diversity in bacterial species. Angew. Chem. Int. Ed. Engl.

[57] Tavender T.J., Halliday N.M., Hardie K.R., Winzer K. (2008). LuxS-independent formation of AI-2 from ribulose-5-phosphate. BMC Microbiol.

[58] Kong P., Tyler B.M., Richardson P.A., Lee B.W., Zhou Z.S., Hong C. (2010). Zoospore interspecific signaling promotes plant infection by *Phytophthora*. BMC Microbiol.

[59] Nichols J.D., Johnson M.R., Chou C.J., Kelly R.M. (2009). Temperature, not LuxS, mediates AI-2 formation in hydrothermal habitats. FEMS Microbiol. Ecol.

[60] Gonzalez J.E., Keshavan N.D. (2006). Messing with bacterial quorum sensing. Microbiol. Mol. Biol. Rev.

[61] Meijler M.M., Hom L.G., Kaufmann G.F., McKenzie K.M., Sun C., Moss J.A., Matsushita M., Janda K.D. (2004). Synthesis and biological validation of a ubiquitous quorum-sensing molecule. Angew. Chem. Int. Ed. Engl.

[62] Lowery C.A., Park J., Kaufmann G.F., Janda K.D. (2008). An unexpected switch in the modulation of AI-2-based quorum sensing discovered through synthetic 4,5-dihydroxy-2,3-pentanedione analogues. J. Am. Chem. Soc.

[63] Tsuchikama K., Zhu J., Lowery C.A., Kaufmann G.F., Janda K.D. (2012). C4-alkoxy-HPD: A potent class of synthetic modulators surpassing nature in AI-2 quorum sensing. J. Am. Chem. Soc.

[64] Tsuchikama K., Lowery C.A., Janda K.D. (2011). Probing autoinducer-2 based quorum sensing: The biological consequences of molecules unable to traverse equilibrium states. J. Org. Chem.

[65] Smith J.A., Wang J., Nguyen-Mau S.M., Lee V., Sintim H.O. (2009). Biological screening of a diverse set of AI-2 analogues in *Vibrio harveyi* suggests that receptors which are involved in synergistic agonism of AI-2 and analogues are promiscuous. Chem. Commun (Camb).

[66] Roy V., Smith J.A., Wang J., Stewart J.E., Bentley W.E., Sintim H.O. (2010). Synthetic analogs tailor native AI-2 signaling across bacterial species. J. Am. Chem. Soc.

[67] Gamby S., Roy V., Guo M., Smith J.A., Wang J., Stewart J.E., Wang X., Bentley W.E., Sintim H.O. (2012). Altering the communication networks of multispecies microbial systems using a diverse toolbox of AI-2 analogues. ACS Chem. Biol.

[68] Guo M., Gamby S., Nakayama S., Smith J., Sintim H.O. (2012). A pro-drug approach for selective modulation of AI-2-mediated bacterial cell-to-cell communication. Sensors (Basel).

[69] Frezza M., Balestrino D., Soulère L., Reverchon S., Queneau Y., Forestier C., Doutheau A. (2006). Synthesis and biological evaluation of the trifluoromethyl analog of (4*S*)-4,5-Dihydroxy-2,3-pentanedione (DPD). Eur. J. Org. Chem.

[70] Ganin H., Tang X., Meijler M.M. (2009). Inhibition of *Pseudomonas aeruginosa* quorum sensing by AI-2 analogs. Bioorg. Med. Chem. Lett.

[71] Rui F., Marques J.C., Miller S.T., Maycock C.D., Xavier K.B., Ventura M.R. (2012). Stereochemical diversity of AI-2 analogs modulates quorum sensing in *Vibrio harveyi* and *Escherichia coli*. Bioorg. Med. Chem.

[72] Semmelhack M.F., Campagna S.R., Federle M.J., Bassler B.L. (2005). An expeditious synthesis of DPD and boron binding studies. Org. Lett.

[73] De Keersmaecker S.C., Varszegi C., van Boxel N., Habel L.W., Metzger K., Daniels R., Marchal K., De Vos D., Vanderleyden J. (2005). Chemical synthesis of (*S*)-4,5-dihydroxy-2,3-pentanedione, a bacterial signal molecule precursor, and validation of its activity in *Salmonella typhimurium*. J. Biol. Chem.

[74] Frezza M., Soulère L., Queneau Y., Doutheau A. (2005). A Baylis–Hillman/ozonolysis route towards (±) 4,5-dihydroxy-2,3-pentanedione (DPD) and analogues. Tetrahedron Lett.

[75] Trost B.M., Malhotra S., Fried B.A. (2009). Magnesium-catalyzed asymmetric direct aldol addition of ethyl diazoacetate to aromatic, aliphatic, and alpha, beta-unsaturated aldehydes. J. Am. Chem. Soc.

[76] Yao W., Wang J. (2003). Direct catalytic asymmetric aldol-type reaction of aldehydes with ethyl diazoacetate. Org. Lett.

[77] Kadirvel M., Stimpson W.T., Moumene-Afifi S., Arsic B., Glynn N., Halliday N., Williams P., Gilbert P., McBain A.J., Freeman S. (2010). Synthesis and bioluminescence-inducing properties of autoinducer (*S*)-4,5-dihydroxypentane-2,3-dione and its enantiomer. Bioorg. Med. Chem. Lett.

[78] Ascenso O.S., Marques J.C., Santos A.R., Xavier K.B., Ventura M.R., Maycock C.D. (2011). An efficient synthesis of the precursor of AI-2, the signalling molecule for inter-species quorum sensing. Bioorg. Med. Chem.

[79] Ng W.L., Bassler B.L. (2009). Bacterial quorum-sensing network architectures. Annu. Rev. Genet.

[80] Neiditch M.B., Federle M.J., Pompeani A.J., Kelly R.C., Swem D.L., Jeffrey P.D., Bassler B.L., Hughson F.M. (2006). Ligand-induced asymmetry in histidine sensor kinase complex regulates quorum sensing. Cell.

[81] Lenz D.H., Mok K.C., Lilley B.N., Kulkarni R.V., Wingreen N.S., Bassler B.L. (2004). The small RNA chaperone Hfq and multiple small RNAs control quorum sensing in *Vibrio harveyi* and *Vibrio cholerae*. Cell.

[82] Herzberg M., Kaye I.K., Peti W., Wood T.K. (2006). YdgG (TqsA) controls biofilm formation in *Escherichia coli* K-12 through autoinducer 2 transport. J. Bacteriol.

[83] Taga M.E., Semmelhack J.L., Bassler B.L. (2001). The LuxS-dependent autoinducer AI-2 controls the expression of an ABC transporter that functions in AI-2 uptake in *Salmonella typhimurium*. Mol. Microbiol.

[84] Miller S.T., Xavier K.B., Campagna S.R., Taga M.E., Semmelhack M.F., Bassler B.L., Hughson F.M. (2004). *Salmonella typhimurium* recognizes a chemically distinct form of the bacterial quorum-sensing signal AI-2. Mol. Cell.

[85] Xavier K.B., Bassler B.L. (2005). Regulation of uptake and processing of the quorum-sensing autoinducer AI-2 in *Escherichia coli*. J. Bacteriol.

[86] Taga M.E., Miller S.T., Bassler B.L. (2003). Lsr-mediated transport and processing of AI-2 in *Salmonella typhimurium*. Mol. Microbiol.

[87] Li J., Attila C., Wang L., Wood T.K., Valdes J.J., Bentley W.E. (2007). Quorum sensing in *Escherichia coli* is signaled by AI-2/LsrR: Effects on small RNA and biofilm architecture. J. Bacteriol.

[88] Wang L., Li J., March J.C., Valdes J.J., Bentley W.E. (2005). luxS-dependent gene regulation in *Escherichia coli* K-12 revealed by genomic expression profiling. J. Bacteriol.

[89] Gonzalez Barrios A.F., Zuo R., Hashimoto Y., Yang L., Bentley W.E., Wood T.K. (2006). Autoinducer 2 controls biofilm formation in *Escherichia coli* through a novel motility quorum-sensing regulator (MqsR, B3022). J. Bacteriol.

[90] Wu M., Tao Y., Liu X., Zang J. (2013). Structural basis for phosphorylated autoinducer-2 modulation of the oligomerization state of the global transcription regulator LsrR from *Escherichia coli*. J. Biol. Chem.

[91] Pereira C.S., de Regt A.K., Brito P.H., Miller S.T., Xavier K.B. (2009). Identification of functional LsrB-like autoinducer-2 receptors. J. Bacteriol.

[92] Ferro A.J., Barrett A., Shapiro S.K. (1976). Kinetic properties and the effect of substrate analogues on 5′-methylthioadenosine nucleosidase from *Escherichia coli*. Biochim. Biophys. Acta.

[93] Singh V., Shi W., Almo S.C., Evans G.B., Furneaux R.H., Tyler P.C., Painter G.F., Lenz D.H., Mee S., Zheng R. (2006). Structure and inhibition of a quorum sensing target from *Streptococcus pneumoniae*. Biochemistry.

[94] Gutierrez J.A., Crowder T., Rinaldo-Matthis A., Ho M.C., Almo S.C., Schramm V.L. (2009). Transition state analogs of 5′-methylthioadenosine nucleosidase disrupt quorum sensing. Nat. Chem. Biol.

[95] Lee J.E., Settembre E.C., Cornell K.A., Riscoe M.K., Sufrin J.R., Ealick S.E., Howell P.L. (2004). Structural comparison of MTA phosphorylase and MTA/AdoHcy nucleosidase explains substrate preferences and identifies regions exploitable for inhibitor design. Biochemistry.

[96] Longshaw A.I., Adanitsch F., Gutierrez J.A., Evans G.B., Tyler P.C., Schramm V.L. (2010). Design and synthesis of potent “sulfur-free” transition state analogue inhibitors of 5′-methylthioadenosine nucleosidase and 5′-methylthioadenosine phosphorylase. J. Med. Chem.

[97] Zhao G., Wan W., Mansouri S., Alfaro J.F., Bassler B.L., Cornell K.A., Zhou Z.S. (2003). Chemical synthesis of *S*-ribosyl-l-homocysteine and activity assay as a LuxS substrate. Bioorg. Med. Chem. Lett.

[98] Alfaro J.F., Zhang T., Wynn D.P., Karschner E.L., Zhou Z.S. (2004). Synthesis of LuxS inhibitors targeting bacterial cell-cell communication. Org. Lett.

[99] Zhu J., Dizin E., Hu X., Wavreille A.-S., Park J., Pei D. (2003). *S*-Ribosylhomocysteinase (LuxS) is a mononuclear iron protein. Biochemistry.

[100] Malladi V.L., Sobczak A.J., Meyer T.M., Pei D., Wnuk S.F. (2011). Inhibition of LuxS by *S*-ribosylhomocysteine analogues containing a [4-aza]ribose ring. Bioorg. Med. Chem.

[101] Shen G., Rajan R., Zhu J., Bell C.E., Pei D. (2006). Design and synthesis of substrate and intermediate analogue inhibitors of *S*-ribosylhomocysteinase. J. Med. Chem.

[102] Wnuk S.F., Robert J., Sobczak A.J., Meyers B.P., Malladi V.L., Zhu J., Gopishetty B., Pei D. (2009). Inhibition of *S*-ribosylhomocysteinase (LuxS) by substrate analogues modified at the ribosyl C-3 position. Bioorg. Med. Chem.

[103] Gopishetty B., Zhu J., Rajan R., Sobczak A.J., Wnuk S.F., Bell C.E., Pei D. (2009). Probing the catalytic mechanism of *S*-ribosylhomocysteinase (LuxS) with catalytic intermediates and substrate analogues. J. Am. Chem. Soc.

[104] Gram L., de Nys R., Maximilien R., Givskov M., Steinberg P., Kjelleberg S. (1996). Inhibitory effects of secondary metabolites from the red alga *delisea pulchra* on swarming motility of *Proteus mirabilis*. Appl. Environ. Microbiol.

[105] Givskov M., de Nys R., Manefield M., Gram L., Maximilien R., Eberl L., Molin S., Steinberg P.D., Kjelleberg S. (1996). Eukaryotic interference with homoserine lactone-mediated prokaryotic signalling. J. Bacteriol.

[106] Ren D., Sims J.J., Wood T.K. (2001). Inhibition of biofilm formation and swarming of *Escherichia coli* by (5*Z*)-4-bromo-5-(bromomethylene)-3-butyl-2(5*H*)-furanone. Environ. Microbiol.

[107] Defoirdt T., Crab R., Wood T.K., Sorgeloos P., Verstraete W., Bossier P. (2006). Quorum sensing-disrupting brominated furanones protect the gnotobiotic brine shrimp *Artemia franciscana* from pathogenic *Vibrio harveyi*, *Vibrio campbellii*, and *Vibrio parahaemolyticus* isolates. Appl. Environ. Microbiol.

[108] Janssens J.C., Steenackers H., Robijns S., Gellens E., Levin J., Zhao H., Hermans K., De Coster D., Verhoeven T.L., Marchal K. (2008). Brominated furanones inhibit biofilm formation by *Salmonella enterica* serovar Typhimurium. Appl. Environ. Microbiol.

[109] Shetye G., Singh N., Gao X., Bandyopadhyay D., Yan A., Luk Y.-Y. (2013). Structures and biofilm inhibition activities of brominated furanones for *Escherichia coli* and *Pseudomonas aeruginosa*. Med. Chem. Comm.

[110] Han X., Lu C. (2009). Biological activity and identification of a peptide inhibitor of LuxS from *Streptococcus suis* serotype 2. FEMS Microbiol. Lett.

[111] Roy V., Fernandes R., Tsao C.-Y., Bentley W.E. (2009). cross species quorum quenching using a native ai-2 processing enzyme. ACS Chem. Biol.

[112] Xue X., Pasparakis G., Halliday N., Winzer K., Howdle S.M., Cramphorn C.J., Cameron N.R., Gardner P.M., Davis B.G., Fernandez-Trillo F. (2011). Synthetic polymers for simultaneous bacterial sequestration and quorum sense interference. Angew. Chem. Int. Ed. Engl.

[113] Li M., Ni N., Chou H.T., Lu C.D., Tai P.C., Wang B. (2008). Structure-based discovery and experimental verification of novel AI-2 quorum sensing inhibitors against *Vibrio harveyi*. Chem. Med. Chem.

[114] Peng H., Cheng Y., Ni N., Li M., Choudhary G., Chou H.T., Lu C.D., Tai P.C., Wang B. (2009). Synthesis and evaluation of new antagonists of bacterial quorum sensing in *Vibrio harveyi*. Chem. Med. Chem.

[115] Ni N., Chou H.T., Wang J., Li M., Lu C.D., Tai P.C., Wang B. (2008). Identification of boronic acids as antagonists of bacterial quorum sensing in *Vibrio harveyi*. Biochem. Biophys. Res. Commun.

[116] Ni N., Choudhary G., Peng H., Li M., Chou H.T., Lu C.D., Gilbert E.S., Wang B. (2009). Inhibition of quorum sensing in *Vibrio harveyi* by boronic acids. Chem. Biol. Drug. Des.

[117] Ni N., Choudhary G., Li M., Wang B. (2008). Pyrogallol and its analogs can antagonize bacterial quorum sensing in *Vibrio harveyi*. Bioorg. Med. Chem. Lett.

[118] Ni N., Choudhary G., Li M., Wang B. (2009). A new phenothiazine structural scaffold as inhibitors of bacterial quorum sensing in *Vibrio harveyi*. Biochem. Biophys. Res. Commun.

[119] Zhu P., Peng H., Ni N., Wang B., Li M. (2012). Novel AI-2 quorum sensing inhibitors in *Vibrio harveyi* identified through structure-based virtual screening. Bioorg. Med. Chem. Lett.

[120] Brackman G., Celen S., Baruah K., Bossier P., Van Calenbergh S., Nelis H.J., Coenye T. (2009). AI-2 quorum-sensing inhibitors affect the starvation response and reduce virulence in several *Vibrio* species, most likely by interfering with LuxPQ. Microbiology.

[121] Niu C., Afre S., Gilbert E.S. (2006). Subinhibitory concentrations of cinnamaldehyde interfere with quorum sensing. Lett. Appl. Microbiol.

[122] Brackman G., Defoirdt T., Miyamoto C., Bossier P., Van Calenbergh S., Nelis H., Coenye T. (2008). Cinnamaldehyde and cinnamaldehyde derivatives reduce virulence in *Vibrio* spp. by decreasing the DNA-binding activity of the quorum sensing response regulator LuxR. BMC Microbiol.

[123] Brackman G., Celen S., Hillaert U., Van Calenbergh S., Cos P., Maes L., Nelis H.J., Coenye T. (2011). Structure-activity relationship of cinnamaldehyde analogs as inhibitors of AI-2 based quorum sensing and their effect on virulence of *Vibrio* spp. PLoS One.

[124] Brackman G., Al Quntar A.A., Enk C.D., Karalic I., Nelis H.J., Van Calenbergh S., Srebnik M., Coenye T. (2013). Synthesis and evaluation of thiazolidinedione and dioxazaborocane analogues as inhibitors of AI-2 quorum sensing in *Vibrio harveyi*. Bioorg. Med. Chem.

[125] Aharoni R., Bronstheyn M., Jabbour A., Zaks B., Srebnik M., Steinberg D. (2008). Oxazaborolidine derivatives inducing autoinducer-2 signal transduction in *Vibrio harveyi*. Bioorg. Med. Chem.

[126] Defoirdt T., Benneche T., Brackman G., Coenye T., Sorgeloos P., Scheie A.A. (2012). A quorum sensing-disrupting brominated thiophenone with a promising therapeutic potential to treat luminescent vibriosis. PLoS One.

[127] Lowery C.A., McKenzie K.M., Qi L., Meijler M.M., Janda K.D. (2005). Quorum sensing in *Vibrio harveyi*: Probing the specificity of the LuxP binding site. Bioorg. Med. Chem. Lett.

[128] Kamaraju K., Smith J., Wang J., Roy V., Sintim H.O., Bentley W.E., Sukharev S. (2011). Effects on membrane lateral pressure suggest permeation mechanisms for bacterial quorum signaling molecules. Biochemistry.

[129] Zhu J., Hixon M.S., Globisch D., Kaufmann G.F., Janda K.D. (2013). Mechanistic insights into the lsrk kinase required for autoinducer-2 quorum sensing activation. J. Am. Chem. Soc.

[130] Furlani R.E., Yeagley A.A., Melander C. (2013). A flexible approach to 1,4-di-substituted 2-aminoimidazoles that inhibit and disperse biofilms and potentiate the effects of beta-lactams against multi-drug resistant bacteria. Eur. J. Med. Chem.

[131] Worthington R.J., Richards J.J., Melander C. (2012). Small molecule control of bacterial biofilms. Org. Biomol. Chem.

[132] Hoiby N., Bjarnsholt T., Givskov M., Molin S., Ciofu O. (2010). Antibiotic resistance of bacterial biofilms. Int. J. Antimicrob. Agents.

[133] Anderson G.G., O’Toole G.A. (2008). Innate and induced resistance mechanisms of bacterial biofilms. Curr. Top. Microbiol. Immunol.

[134] Stewart P.S., Costerton J.W. (2001). Antibiotic resistance of bacteria in biofilms. Lancet.

[135] Costerton J.W., Stewart P.S., Greenberg E.P. (1999). Bacterial biofilms: A common cause of persistent infections. Science.

[136] Mah T.F., O’Toole G.A. (2001). Mechanisms of biofilm resistance to antimicrobial agents. Trends Microbiol.

[137] Brackman G., Cos P., Maes L., Nelis H.J., Coenye T. (2011). Quorum sensing inhibitors increase the susceptibility of bacterial biofilms to antibiotics *in vitro* and *in vivo*. Antimicrob. Agents Chemother.

[138] Frezza M., Soulere L., Balestrino D., Gohar M., Deshayes C., Queneau Y., Forestier C., Doutheau A. (2007). Ac2-DPD, the bis-(*O*)-acetylated derivative of 4,5-dihydroxy-2,3-pentanedione (DPD) is a convenient stable precursor of bacterial quorum sensing autoinducer AI-2. Bioorg. Med. Chem. Lett.

[139] Soni K.A., Jesudhasan P., Cepeda M., Widmer K., Jayaprakasha G.K., Patil B.S., Hume M.E., Pillai S.D. (2008). Identification of ground beef-derived fatty acid inhibitors of autoinducer-2-based cell signaling. J. Food Prot.

[140] Widmer K.W., Soni K.A., Hume M.E., Beier R.C., Jesudhasan P., Pillai S.D. (2007). Identification of poultry meat-derived fatty acids functioning as quorum sensing signal inhibitors to autoinducer-2 (AI-2). J. Food Sci.

[141] Sun J., Daniel R., Wagner-Dobler I., Zeng A.P. (2004). Is autoinducer-2 a universal signal for interspecies communication: A comparative genomic and phylogenetic analysis of the synthesis and signal transduction pathways. BMC Evol. Biol.

[142] Winzer K., Hardie K.R., Williams P. (2003). LuxS and autoinducer-2: Their contribution to quorum sensing and metabolism in bacteria. Adv. Appl. Microbiol.

[143] Diggle S.P., Gardner A., West S.A., Griffin A.S. (2007). Evolutionary theory of bacterial quorum sensing: When is a signal not a signal?. Philos. Trans. R. Soc. Lond. B.

[144] Hegde M., Englert D.L., Schrock S., Cohn W.B., Vogt C., Wood T.K., Manson M.D., Jayaraman A. (2011). Chemotaxis to the quorum-sensing signal AI-2 requires the Tsr chemoreceptor and the periplasmic LsrB AI-2-binding protein. J. Bacteriol.

